# Supplementary material for: Exact simulation of pigment-protein complexes unveils vibronic renormalization of electronic parameters in ultrafast spectroscopy
Source: Nat Commun. 2022 May 25;13:2912. doi: 10.1038/s41467-022-30565-4 (PMC9133012; doi:10.1038/s41467-022-30565-4)
Supplement: Supplementary file 1 — Supplementary Information [file 41467_2022_30565_MOESM1_ESM.pdf]

Supplementary Information: Exact Simulation of Pigment-Protein Complexes Unveils Vibronic Renormalization of Electronic Parameters in Ultrafast Spectroscopy

M. B. Plenio et al.

Supplementary Figures:

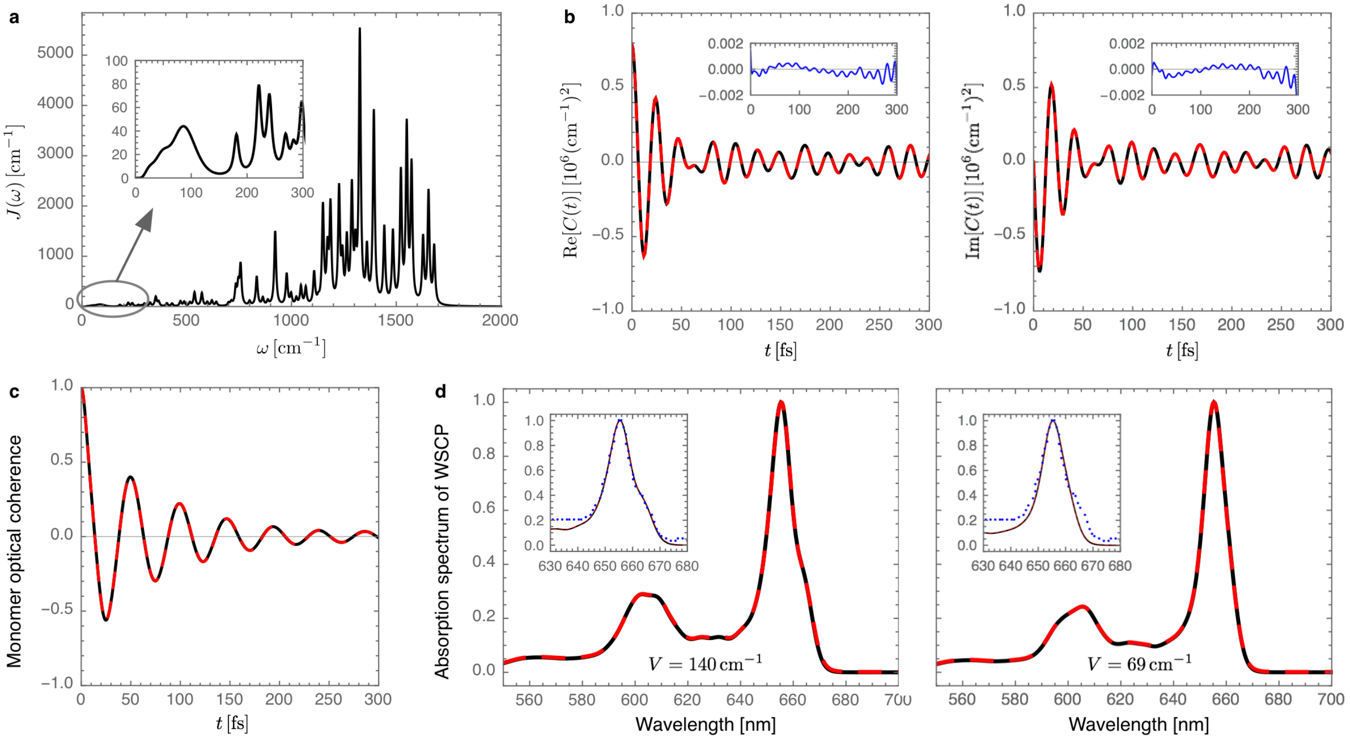

Supplementary Figure 1. (**a**) The experimentally estimated phonon spectral density $J(\omega)$ of WSCP consists of three log-normal functions at low frequencies $\omega \lesssim 150\,\mathrm{cm}^{-1}$ and 55 high-frequency underdamped modes modelled by Lorentzian functions with a vibrational damping rate of $(1\,\mathrm{ps})^{-1}$. The inset shows the low energy part of the spectral density in detail. (**b**) The real and imaginary parts of the bath correlation function (BCF) $C(t)$ at $T = 77\,\mathrm{K}$, shown in black, are fitted up to 300 fs with the sum of 13 exponentials as shown in red dashed lines. The insets show the difference between target BCF and the fitting function, demonstrating that the fitting error is three orders of magnitude smaller than the amplitudes of the target BCF. (**c**) The dynamics of the real part of the optical coherence of a two-level monomer in the absence of static disorder. The simulated HEOM results, shown in red, are well matched to the analytical solution shown in black: the site energy of the monomer is taken to be zero for better visibility. The imaginary part of the coherence computed by HEOM is also well matched to the analytical solution (not shown here). (**d**) The absorption spectra of WSCP homodimers with $V = 140\,\mathrm{cm}^{-1}$ or $V = 69\,\mathrm{cm}^{-1}$. The simulated HEOM results shown in red are well matched to the TEDOPA results shown in black. The simulated optical coherence of the dimer system decays within 300 fs mainly due to the static disorder (not shown here), implying that the fitting of the BCF up to 300 fs is sufficient for HEOM simulations. Experimentally measured absorption spectrum of WSCP is shown in blue as a guide for the eye. For $V = 140\,\mathrm{cm}^{-1}$ ($V = 69\,\mathrm{cm}^{-1}$), the mean site energies $\langle \varepsilon_1 \rangle = \langle \varepsilon_2 \rangle$ are taken to be 631.5 nm (629.8 nm) and the standard deviation of the Gaussian static disorder is $80\,\mathrm{cm}^{-1}$ ($72\,\mathrm{cm}^{-1}$). The angle between transition dipole moments of monomers is taken to be 39°.

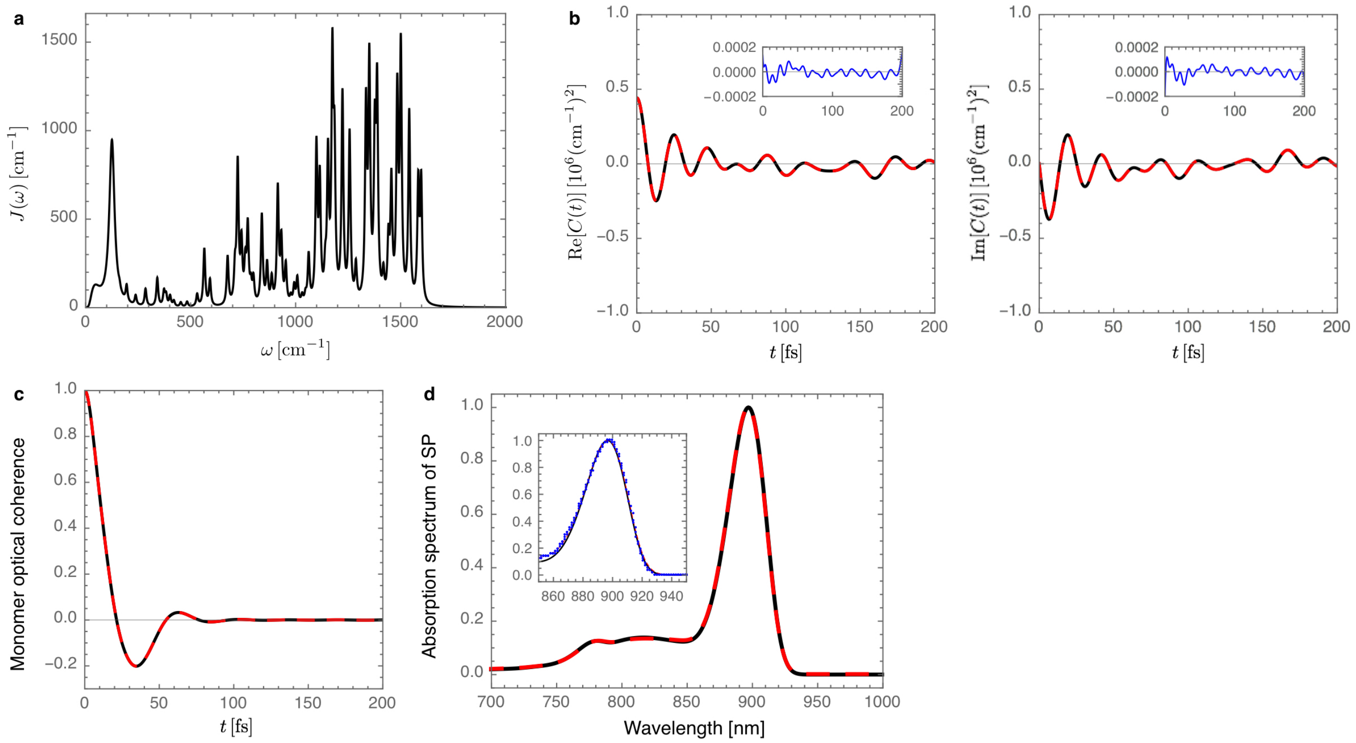

Supplementary Figure 2. (**a**) Experimentally estimated phonon spectral density $J(\omega)$ of the special pair in bacterial reaction centers. (**b**) The bath correlation function (BCF) at $T = 5\,\mathrm{K}$, denoted by $C(t)$, is shown in black, which is fitted up to 200 fs with the sum of 16 exponentials as shown in red dashed lines. The difference between target BCF and fitting function is shown in blue, demonstrating that the fitting error is three orders of magnitude smaller than the amplitudes of the target BCF. (**c**) The dynamics of the real part of the optical coherence of a two-level monomer in the absence of static disorder. The simulated HEOM results shown in red are well matched to the analytical solution shown in black: the site energy of the monomer is taken to be zero for better visibility. (**d**) The absorption spectra of the special pair. The simulated HEOM results shown in red are well matched to the TEDOPA results shown in black. Experimentally measured absorption spectra of bacterial reaction centers are shown in blue as a guide for the eye. The mean site energies of sites 1 and 2 are taken to be 814.2 nm and 835.7 nm, respectively, with the detuning of $\langle \varepsilon_1 - \varepsilon_2 \rangle = 315\,\mathrm{cm}^{-1}$. The standard deviation of local static disorder is taken to be $105\,\mathrm{cm}^{-1}$. The angle between transition dipole moments of monomers is taken to be 143°.

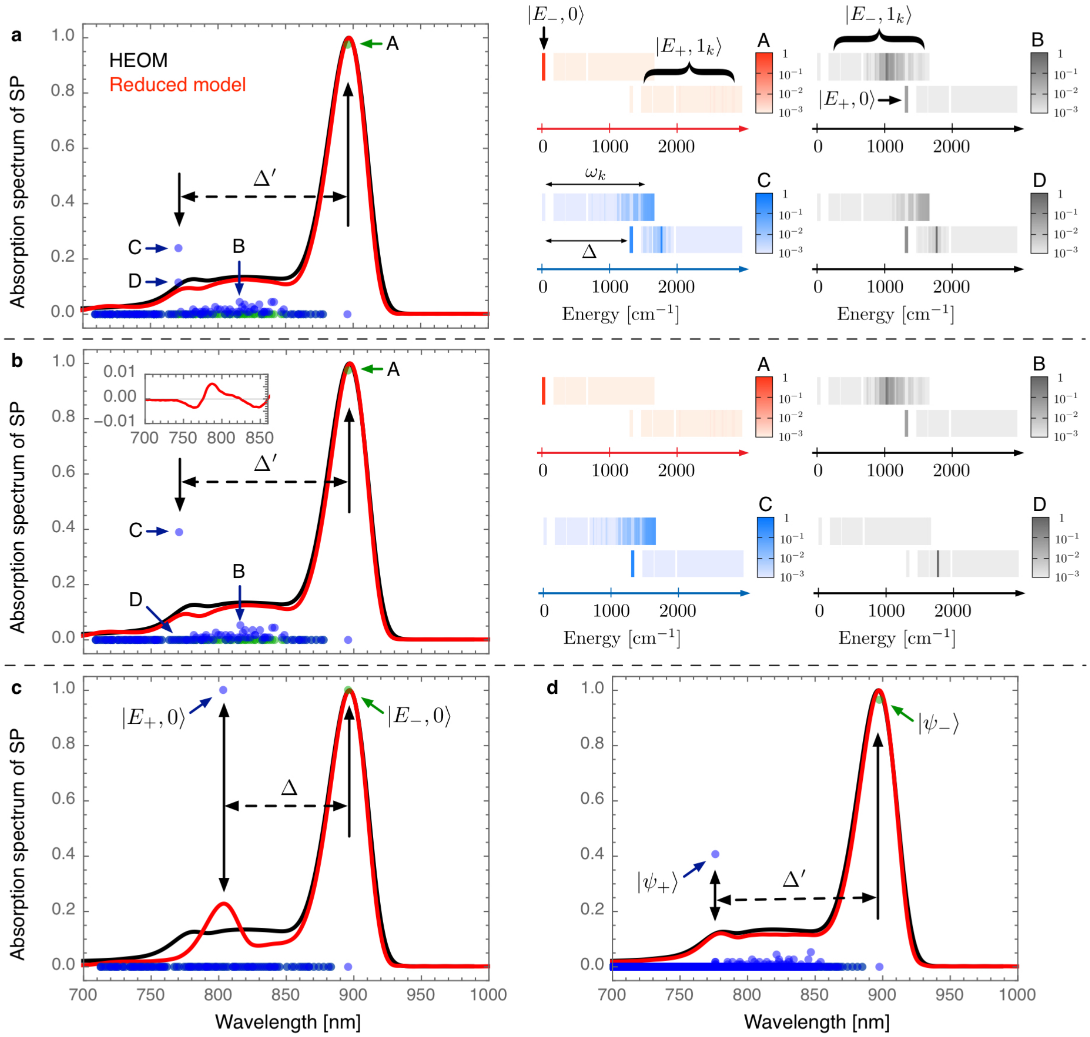

Supplementary Figure 3. (**a**) Comparison of absorption line shapes of the special pair in bacterial reaction centers computed by HEOM (black) and reduced vibronic model (red). For each vibronic eigenstate of $\tilde{H}$, its transition energy from global ground state $|g, 0\rangle$ and the fidelity with a vibrationally cold exciton state $|E_-, 0\rangle$ ($|E_+, 0\rangle$) are shown as a green (blue) dot. For vibronic eigenstates A-D, the fidelity with vibrationally cold $|E_\pm, 0\rangle$ and vibrationally hot $|E_\pm, 1_k\rangle$ states is displayed to visualize the presence of multi-mode vibronic mixing. (**b**) Reduced model results without higher-order interaction terms. The difference between approximate absorption line shapes obtained by reduced models with and without the higher-order couplings is shown in the inset. (**c**) Reduced model results without off-diagonal vibronic couplings. (**d**) Reduced model results where double vibrational excitations are considered: in (**a**)-(**c**), single vibrational excitation subspace is considered.

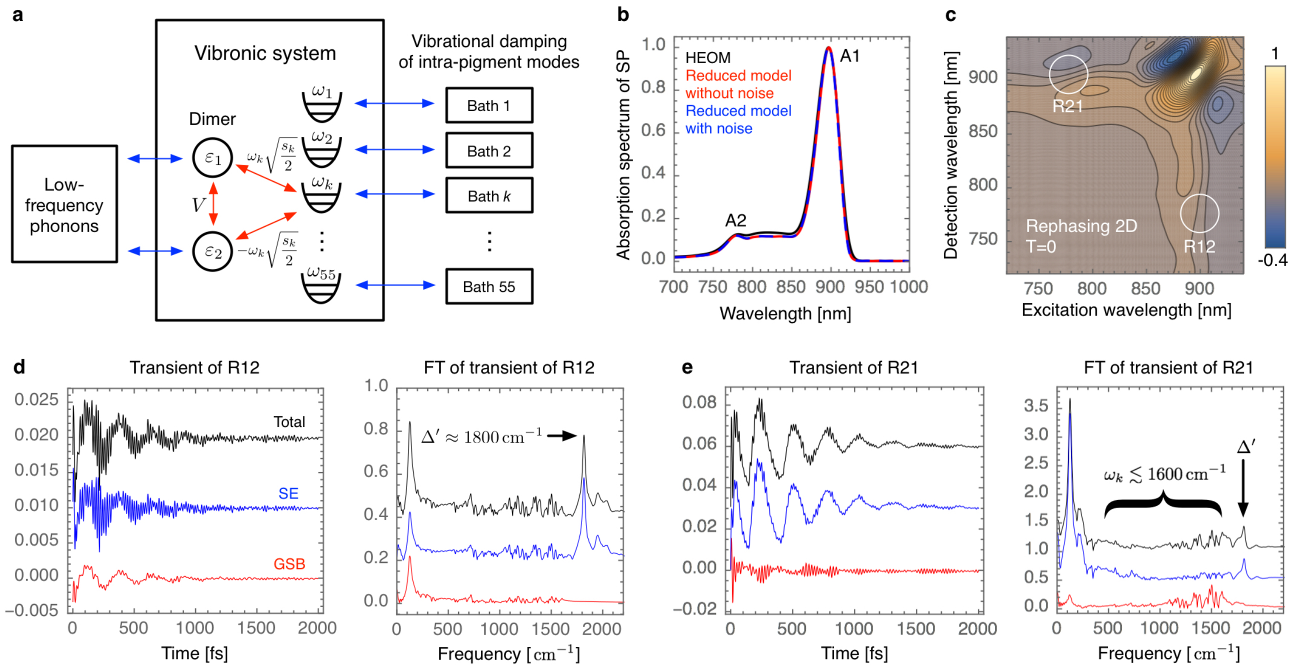

Supplementary Figure 4. (**a**) Schematic representation of a vibronic model in the presence of noise. The vibronic system is characterized by the interaction between electronic states of a dimer and 55 oscillators associated with the relative motions of intra-pigment modes. The coupling between electronic states and low-frequency phonons induces electronic dephasing. In addition, each oscillator is coupled to an independent secondary bath, inducing vibrational damping. (**b**) Absorption spectra of SP computed by HEOM and reduced vibronic models without and with noise are shown in black, red and blue, respectively, where double vibrational excitation subspace is considered. (**c**) Rephasing 2D spectra at waiting time $T = 0$. (**d**) 2D signals at a cross-peak R12, marked in (**c**), and corresponding Fourier transformation where total (GSB+SE), ground state (GSB) and excited state (SE) signals are shown in black, red and blue, respectively. Note that excited state signals as well as total 2D signals, namely the sum of ground and excited state signals, are dominated by vibronic coherence $|\psi_+\rangle\langle\psi_-|$, leading to 2D oscillations at $\Delta' \approx 1800\,\mathrm{cm}^{-1}$. (**e**) 2D signals at a cross-peak R21, marked in (**c**). Note that excited state signals are dominated by 2D oscillations at $\Delta' \approx 1800\,\mathrm{cm}^{-1}$, while ground state coherences are dominated by multiple components with frequencies $\omega_k \lesssim 1600\,\mathrm{cm}^{-1}$, which are lower than $\Delta' \approx 1800\,\mathrm{cm}^{-1}$. The offset values of transients and FTs are shifted for better visibility.

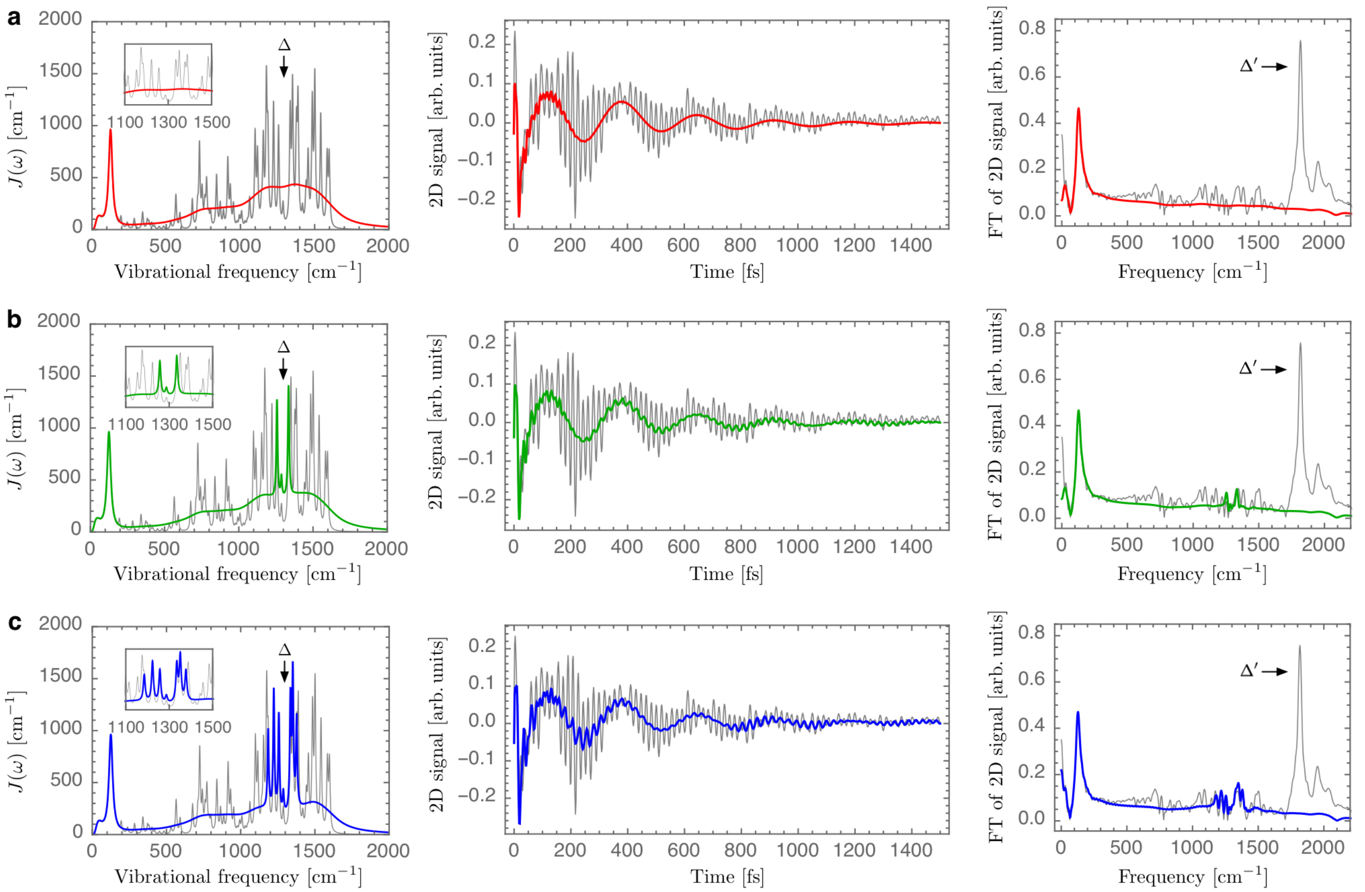

Supplementary Figure 5. (**a**) Fully coarse-grained spectral density is shown in red where all the 55 intra-pigment modes are modelled by broad Lorentzian spectral densities with a uniform width of $\gamma_k = (50\,\mathrm{fs})^{-1}$. Experimentally estimated phonon spectral density of SP is shown in grey where $\gamma_k = (1\,\mathrm{ps})^{-1}$ for all the 55 modes. Transient of a cross-peak R12 (SE component only, see Supplementary Figure 4c and d) and its frequency spectrum, computed based on the fully coarse-grained spectral density, are shown in red where long-lived oscillations at $\Delta' \approx 1800\,\mathrm{cm}^{-1}$ frequency are absent. (**b**) Partially coarse-grained spectral density is shown in green where the three intra-pigment modes whose frequencies are near-resonant with the excitonic splitting $\Delta \approx 1300\,\mathrm{cm}^{-1}$ of the SP are modelled by narrow Lorentzian spectral densities with a width of $\gamma_k = (1\,\mathrm{ps})^{-1}$, while all the other intra-pigment modes are modelled by the broad Lorentzian functions with $\gamma_k = (50\,\mathrm{fs})^{-1}$. The transient of the cross-peak R12 includes long-lived oscillations at the vibrational frequencies $\omega_k \approx \Delta$ of the near-resonant modes, as shown in green, but long-lived $\Delta' \approx 1800\,\mathrm{cm}^{-1}$ oscillations are absent. (**c**) Similar features of 2D spectra are obtained when seven near-resonant intra-pigment modes are modelled by the narrow Lorentzian spectral densities, as shown in blue. We note that in 2D simulations, static disorder is considered where the standard deviation of a Gaussian distribution of the excitonic splitting is taken to be $\sqrt{2}\sigma \approx 150\,\mathrm{cm}^{-1}$.

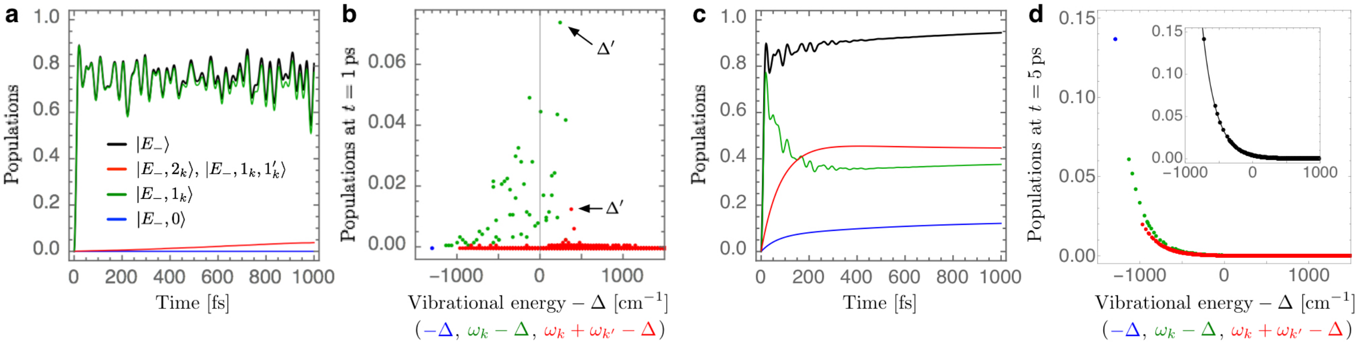

Supplementary Figure 6. (**a**) Population dynamics of the exciton states of SP coupled to undamped 55 intra-pigment modes (relative motion modes). The vibronic coupling to low-frequency phonon environments is ignored, so that the population dynamics is coherent. The total populations of vibrationally cold ($|E_-,0\rangle$), singly excited ($|E_-,1_k\rangle$), and doubly excited states ($|E_-,2_k\rangle$ and $|E_-,1_k,1_{k'}\rangle$) of a lower-energy exciton state $|E_-\rangle$ are shown in blue, green, and red, respectively, and the total population of the lower-energy exciton states is shown in black. (**b**) The populations of $|E_-,0\rangle$, $|E_-,1_k\rangle$, $|E_-,2_k\rangle$ and $|E_-,1_k,1_{k'}\rangle$ at time $t=1\,\mathrm{ps}$ are shown in black, green, red dots, respectively, as a function of the difference in vibrational energy and excitonic splitting Δ. (**c**) The population dynamics in the presence of the Lindblad noise induced by vibrational damping of the intra-pigment modes and the vibronic couplings to the low-frequency phonon environments at room temperature ($T=293\,\mathrm{K}$). (**d**) The populations of $|E_-,0\rangle$, $|E_-,1_k\rangle$, $|E_-,2_k\rangle$ and $|E_-,1_k,1_{k'}\rangle$ at time $t=5\,\mathrm{ps}$ in the presence of the noise. In the inset, the populations of vibronic eigenstates at time $t=5\,\mathrm{ps}$ are shown in black dots as a function of the difference in vibronic energies and excitonic splitting, which are well matched to the Boltzmann distribution of a thermal state at room temperature, shown in black line.

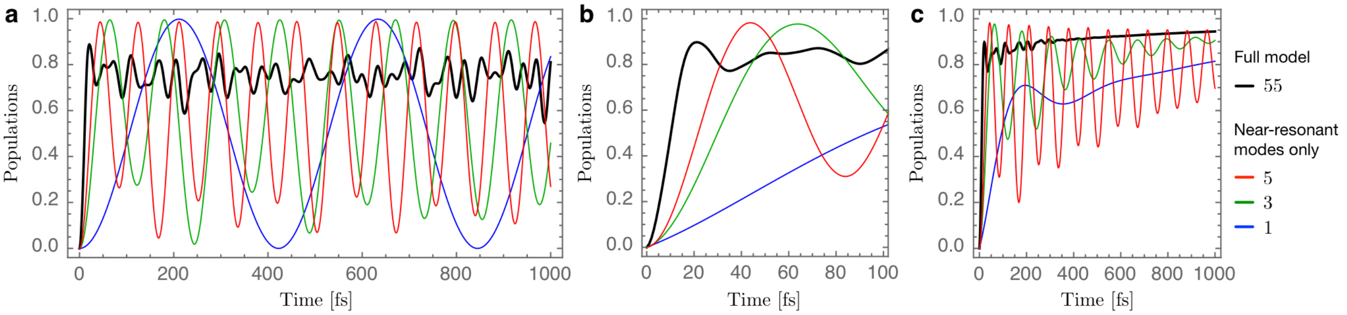

Supplementary Figure 7. (**a**) Population dynamics of a lower-energy exciton state $|E_-\rangle$ of the SP when excitons are coupled to 55 intra-pigment modes (see black line) or coupled only to $M=1,3,5$ intra-pigment modes near-resonant with the excitonic splitting $\Delta\approx 1300\,\mathrm{cm}^{-1}$ of the SP (see blue, green, red lines, respectively). Here the Lindblad noise induced by vibrational damping of the intra-pigment modes and the vibronic couplings to low-frequency phonon environments at room temperature is neglected. (**b,c**) The population dynamics of $|E_-\rangle$ in the presence of the Lindblad noise.

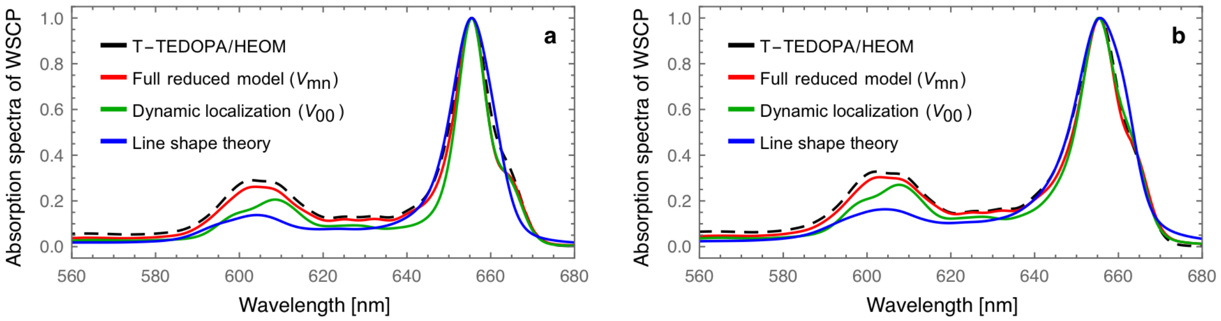

Supplementary Figure 8. (**a**,**b**) For different spectral densities of WSCP, $(J_l^{\mathrm{WSCP}}(\omega) + J_h(\omega), J_l^{\mathrm{B777}}(\omega) + J_h(\omega))$, numerically exact absorption spectra of WSCP are shown in black, which are well matched to approximate results obtained by a full reduced vibronic model taking into account all V_{mn} couplings, shown in red. Approximate absorption spectra obtained by a reduced model where all the V_{mn} couplings are ignored except for V_{00} (dynamic localization) are shown in green. Approximate results computed by the conventional line shape theory are shown in blue where the full environmental spectral density $J(\omega)$ including 55 intrapigment vibrational modes is considered.

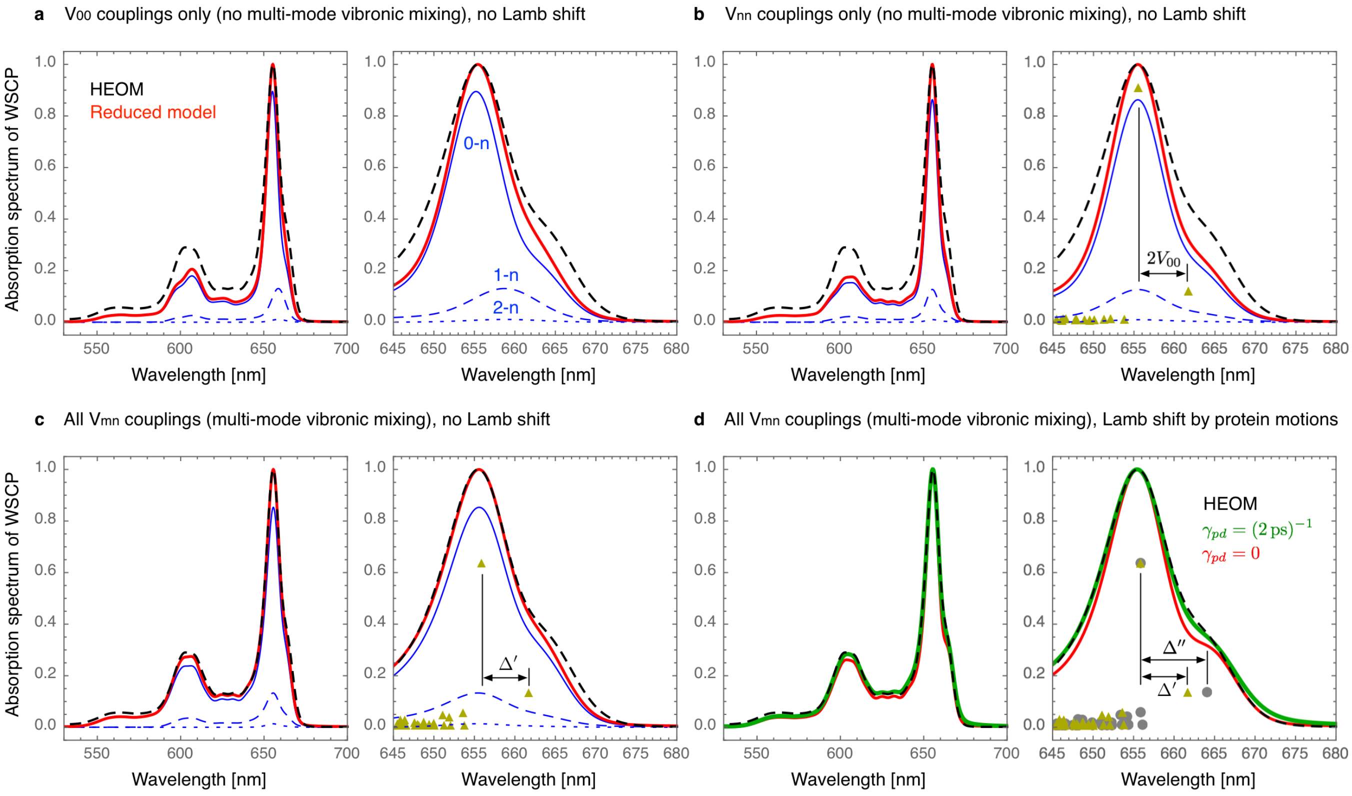

Supplementary Figure 9. (**a**) Numerically exact absorption spectra of WSCP at $T = 77\,\mathrm{K}$ shown in a black dashed line, and approximate absorption line shape computed by numerical diagonalisation of vibronic Hamiltonian $H_0' + V_{00}(|\varepsilon_1, 0\rangle\langle\varepsilon_2, 0| + h.c.)$, shown in a red solid line. The total approximate absorption line shape is a sum of 0-n, 1-n and 2-n transitions shown in blue solid, blue dashed and blue dotted lines, respectively. (**b**) Approximate absorption line shape obtained by numerical diagonalisation of $H_0' + H_I^{(nn)}$ where $H_I^{(nn)}$ contains the couplings between local electronic excitations with identical vibrational states. The transition dipole strengths of 0-n transitions are displayed in yellow triangles as a function of transition energies. (**c**) Approximate absorption spectra based on the full Hamiltonian $H_0' + H_I'$ including the couplings amongst 0-0 and 0-1 transitions. (**d**) Approximate absorption spectra based on $H_0' + H_I' + H_{LS}$ including Lamb shift H_{LS}. Approximate results in the absence and presence of an empirical pure dephasing rate, $\gamma_{pd} = 0$ and $\gamma_{pd} = (2\,\mathrm{ps})^{-1}$, are shown in red and green solid lines, respectively. The transition dipole strengths and energies of 0-n transitions in the presence of the Lamb shift are shown in grey dots.

Supplementary Tables:

Supplementary Table 1. Vibrational frequencies ω_k and Huang-Rhys factors s_k of 55 intra-pigment modes of WSCP consisting of Chl$b$ pigments[1].

k	1	2	3	4	5	6	7	8	9	10
ω_k [cm^{-1}]	181	221	240	269	283	298	325	352	366	405
s_k	0.0173	0.0246	0.0182	0.0064	0.0036	0.0104	0.0112	0.0249	0.0112	0.0061
k	11	12	13	14	15	16	17	18	19	20
ω_k [cm^{-1}]	430	470	488	515	537	572	598	620	641	700
s_k	0.0050	0.0075	0.0061	0.0045	0.0157	0.0132	0.0036	0.0047	0.0033	0.0019
k	21	22	23	24	25	26	27	28	29	30
ω_k [cm^{-1}]	713	734	746	757	800	834	863	887	922	977
s_k	0.0025	0.0107	0.0112	0.0229	0.0022	0.0140	0.0033	0.0019	0.0291	0.0110
k	31	32	33	34	35	36	37	38	39	40
ω_k [cm^{-1}]	998	1023	1045	1068	1108	1128	1150	1172	1186	1227
s_k	0.0036	0.0022	0.0056	0.0050	0.0087	0.0011	0.0244	0.0121	0.0226	0.0249
k	41	42	43	44	45	46	47	48	49	50
ω_k [cm^{-1}]	1243	1264	1288	1305	1326	1360	1393	1443	1484	1522
s_k	0.0090	0.0126	0.0224	0.0093	0.0509	0.0093	0.0328	0.0121	0.0107	0.0185
k	51	52	53	54	55					
ω_k [cm^{-1}]	1550	1573	1628	1654	1681					
s_k	0.0241	0.0182	0.0081	0.0135	0.0067					

Supplementary Table 2. Vibrational frequencies ω_k and Huang-Rhys factors s_k of 55 intra-pigment modes of BChl$a$ pigments[2].

k	1	2	3	4	5	6	7	8	9	10
ω_k [cm^{-1}]	161	195	238	285	341	373	383	402	420	453
s_k	0.0150	0.0400	0.0160	0.0200	0.0230	0.0100	0.0070	0.0060	0.0030	0.0020
k	11	12	13	14	15	16	17	18	19	20
ω_k [cm^{-1}]	483	531	565	592	676	711	724	742	760	772
s_k	0.0020	0.0040	0.0170	0.0070	0.0100	0.0060	0.0250	0.0100	0.0060	0.0120
k	21	22	23	24	25	26	27	28	29	30
ω_k [cm^{-1}]	787	799	839	864	886	915	932	953	977	993
s_k	0.0026	0.0036	0.0120	0.0050	0.0030	0.0130	0.0068	0.0040	0.0007	0.0017
k	31	32	33	34	35	36	37	38	39	40
ω_k [cm^{-1}]	1008	1031	1047	1062	1099	1115	1141	1154	1175	1185
s_k	0.0024	0.0007	0.0007	0.0040	0.0120	0.0090	0.0020	0.0100	0.0160	0.0090
k	41	42	43	44	45	46	47	48	49	50
ω_k [cm^{-1}]	1223	1257	1287	1335	1351	1377	1388	1418	1442	1456
s_k	0.0130	0.0100	0.0020	0.0100	0.0120	0.0076	0.0100	0.0012	0.0026	0.0052
k	51	52	53	54	55					
ω_k [cm^{-1}]	1484	1501	1541	1584	1598					
s_k	0.0087	0.0104	0.0075	0.0044	0.0044					

Supplementary Notes:

Supplementary Note 1. Vibronic Hamiltonian and Polaron Transformation

Here we present the vibronic Hamiltonian that describes the interaction between excitons and vibrational modes in photosynthetic complexes in the exciton basis. This serves to define off-diagonal vibronic couplings that play an essential role in the multi-mode vibronic mixing discussed in the main text.

For simplicity, we consider an excitonically coupled dimer where the electronic excitation of each site is coupled to a local vibrational environment. The total Hamiltonian is modelled by $H = H_e + H_v + H_{e-v}$. The electronic Hamiltonian H_e is characterized by on-site energies ε_i and inter-site electronic coupling V

$$H_e = \sum_{i=1}^{2} \varepsilon_i \left|\varepsilon_i\right\rangle \left\langle\varepsilon_i\right| + V(\left|\varepsilon_1\right\rangle \left\langle\varepsilon_2\right| + \left|\varepsilon_2\right\rangle \left\langle\varepsilon_1\right|). \tag{1}$$

The vibrational environments are modelled by quantum harmonic oscillators

$$H_v = \sum_{i=1}^{2} \sum_k \omega_k b_{i,k}^\dagger b_{i,k}, \tag{2}$$

where $b_{i,k}$ describes a vibrational mode with frequency ω_k, which is locally coupled to site i. The coupling of the electronic degrees of freedom to their respective vibrational environment is described by

$$H_{e-v} = \sum_{i=1}^{2} \left|\varepsilon_i\right\rangle \left\langle\varepsilon_i\right| \sum_k \omega_k \sqrt{s_k}(b_{i,k} + b_{i,k}^\dagger), \tag{3}$$

where the vibronic coupling strength is quantified by a Huang-Rhys factor s_k. To identify the origin of the multi-mode vibronic mixing, we define $B_k = \frac{1}{\sqrt{2}}(b_{1,k} + b_{2,k})$ and $b_k = \frac{1}{\sqrt{2}}(b_{1,k} - b_{2,k})$, describing the center-of-mass and relative motions, respectively, of the local vibrational modes $b_{1,k}$ and $b_{2,k}$ with identical frequency ω_k[3]. B_k and b_k satisfy the bosonic commutation relations and the total Hamiltonian can be expressed as $H = H_e + H_c + H_r$, where

$$H_c = \sum_k \omega_k B_k^\dagger B_k + \mathbb{1}^{(\varepsilon)} \sum_k \omega_k \sqrt{s_k/2}(B_k + B_k^\dagger), \tag{4}$$

$$H_r = \sum_k \omega_k b_k^\dagger b_k + \sigma_z^{(\varepsilon)} \sum_k \omega_k \sqrt{s_k/2}(b_k + b_k^\dagger), \tag{5}$$

with $\mathbb{1}^{(\varepsilon)} = \left|\varepsilon_1\right\rangle \left\langle\varepsilon_1\right| + \left|\varepsilon_2\right\rangle \left\langle\varepsilon_2\right|$ and $\sigma_z^{(\varepsilon)} = \left|\varepsilon_1\right\rangle \left\langle\varepsilon_1\right| - \left|\varepsilon_2\right\rangle \left\langle\varepsilon_2\right|$. Note that the center-of-mass modes B_k are coupled to both electronic excited states $\left|\varepsilon_1\right\rangle$ and $\left|\varepsilon_2\right\rangle$ with the same coupling strength and phase. This implies that the coupling to the center-of-mass motion, described by H_c, does not affect electronic dynamics within the single excitation manifold. Instead, it induces dephasing of optical coherences between electronic ground and excited states, which broadens the absorption line shapes. The contribution of the center-of-mass motion to absorption line width can be taken into account analytically, as described in Supplementary Note 4.

The vibronic coupling to the relative motion of vibrational modes, described by H_r, affects electronic dynamics in the single excitation sector. Here we describe H_r in terms of exciton states $\left|E_\pm\right\rangle$ that diagonalize the electronic Hamiltonian $H_e = E_+ \left|E_+\right\rangle \left\langle E_+\right| + E_- \left|E_-\right\rangle \left\langle E_-\right|$ and are parameterized by

$$\left|E_+\right\rangle = \cos(\theta/2) \left|\varepsilon_1\right\rangle + \sin(\theta/2) \left|\varepsilon_2\right\rangle, \tag{6}$$

$$\left|E_-\right\rangle = -\sin(\theta/2) \left|\varepsilon_1\right\rangle + \cos(\theta/2) \left|\varepsilon_2\right\rangle, \tag{7}$$

where $\tan(\theta) = 2V/(\varepsilon_1 - \varepsilon_2)$ and $\Delta = E_+ - E_- = \sqrt{(\varepsilon_1 - \varepsilon_2)^2 + 4V^2}$ denoting an excitonic splitting. In the exciton basis, H_r is expressed as

$$H_r = \sum_k \omega_k b_k^\dagger b_k + \sum_{i,j} A_{ij} \left|E_i\right\rangle \left\langle E_j\right| \sum_k \omega_k \sqrt{s_k/2}(b_k^\dagger + b_k), \tag{8}$$

where $A_{ij} = \langle E_i|\varepsilon_1\rangle\langle\varepsilon_1|E_j\rangle - \langle E_i|\varepsilon_2\rangle\langle\varepsilon_2|E_j\rangle$, leading to $A_{++} = -A_{--} = \cos(\theta)$ and $A_{+-} = A_{-+} = -\sin(\theta)$. We call the terms proportional to $|E_i\rangle\langle E_i|$ diagonal vibronic couplings, and the other terms proportional to $|E_i\rangle\langle E_j|$ with $i \neq j$ off-diagonal vibronic couplings.

We note that the diagonal vibronic couplings describe the shift of the equilibrium positions of the vibrational modes b_k depending on electronic states. This can be seen more clearly in a displaced vibrational basis. We decompose the total Hamiltonian into two parts $H = H_0 + H_I$ where H_0 includes the diagonal vibronic couplings

$$H_0 = H_e + H_c + \sum_k \omega_k b_k^\dagger b_k + \cos(\theta)\sigma_z \sum_k \omega_k \sqrt{s_k/2}(b_k^\dagger + b_k), \tag{9}$$

with $\sigma_z = |E_+\rangle\langle E_+| - |E_-\rangle\langle E_-|$, while H_I consists of the off-diagonal vibronic couplings

$$H_I = -\sin(\theta)\sigma_x \sum_k \omega_k \sqrt{s_k/2}(b_k^\dagger + b_k). \tag{10}$$

with $\sigma_x = |E_+\rangle\langle E_-| + |E_-\rangle\langle E_+|$. By applying a unitary operator $U = |g\rangle\langle g| + |E_+\rangle\langle E_+|D_\theta + |E_-\rangle\langle E_-|D_\theta^\dagger$ with $D_\theta = \exp(\cos(\theta)\sum_k \sqrt{\frac{s_k}{2}}(b_k^\dagger - b_k))$ to the total Hamiltonian H (the polaron transformation), it can be shown that H_0 is diagonalised as the diagonal vibronic couplings are reduced to the global energy-level shift of exciton states which is proportional to the reorganization energy $\sum_k \omega_k s_k$

$$UH_0U^\dagger = H_e + H_c + \sum_k \omega_k b_k^\dagger b_k - \sum_i |E_i\rangle\langle E_i| \cos^2(\theta) \sum_k \omega_k \frac{s_k}{2},$$

while H_I describes the vibronic mixing between exciton states mediated by vibrational modes b_k

$$UH_IU^\dagger = -\sin(\theta)\,|E_+\rangle\langle E_-|\, D_\theta \sum_k \omega_k \sqrt{s_k/2}(b_k^\dagger + b_k) D_\theta + h.c., \tag{11}$$

where *h.c.* stands for Hermitian conjugate. These off-diagonal couplings are treated perturbatively in the second order cumulant expansion technique[4], which has been employed to compute optical responses of multi-site systems approximately. To fully take into account the off-diagonal vibronic couplings in simulations, one needs to employ numerically exact methods, such as TEDOPA and HEOM discussed in Supplementary Note 3 and Supplementary Note 4, respectively.

Supplementary Note 2. Perturbative Description of Multi-Mode Vibronic Splitting without Polaron Transformation

In the main text, we show how the shift of an excitonic splitting Δ to a vibronic splitting Δ' induced by a multi-mode vibronic mixing is described by second order perturbation theory in the polaron basis. Here we show that a qualitatively similar vibronic energy renormalization can be derived in the regular basis without the polaron transformation by using the second order perturbation theory.

As shown in Supplementary Note 1, the exciton states are mixed with the relative motions of the local vibrational modes and the center-of-mass motions of the local modes do not modulate the energy-gap between exciton states. Therefore we decompose the Hamiltonian of the excitons coupled to the relative motion modes b_k into two parts $H_0 = \sum_i E_i |E_i\rangle\langle E_i| + \sum_k \omega_k b_k^\dagger b_k$ and $H_I = \sigma_z^{(\varepsilon)} \sum_k \omega_k \sqrt{s_k/2}(b_k + b_k^\dagger)$ with $\sigma_z^{(\varepsilon)} = |\varepsilon_1\rangle\langle\varepsilon_1| - |\varepsilon_2\rangle\langle\varepsilon_2|$ where H_I is considered a perturbation term. The eigenstates of H_0 include $|E_+, 0\rangle$, $|E_+, 1_k\rangle$, $|E_-, 0\rangle$, $|E_-, 1_k\rangle$ where $|0\rangle$ denotes a global vibrational ground state where all the modes are in their vibrational ground states, and $|1_k\rangle$ represents a singly excited vibrational state where only the k-th mode is singly excited and all the other modes are in their vibrational ground states. Using the second order perturbation theory, it can be shown that the excitonic splitting $\Delta = E_+ - E_-$ between $|E_+, 0\rangle$ and $|E_-, 0\rangle$ is shifted to a vibronic splitting in the form

$$\Delta' = \Delta \left(1 + \frac{4V^2}{\Delta^2} \sum_k \frac{s_k \omega_k^2}{\Delta^2 - \omega_k^2}\right), \tag{12}$$

which is qualitatively similar to the vibronic splitting estimated in the polaron basis in the sense that $\Delta' < \Delta$ if $\Delta < \omega_k$, and $\Delta' > \Delta$ if $\Delta > \omega_k$ due to the dependence of Δ' on $\Delta^2 - \omega_k^2$. Note that when the total Huang-Rhys factor $\sum_k s_k$ with $s_k \geq 0$ is sufficiently small, namely in the weak vibronic coupling limit, the vibronic splittings Δ' estimated in the regular and polaron bases coincide, as $\alpha = \exp(-2\cos^2(\theta)\sum_k s_k) \to 1$ (see Eq. (9) in the main text).

Supplementary Note 3. TEDOPA

The goal of this work is the numerical evaluation of absorption spectra in the presence of a structured environment as well as energetic and orientational disorder of the sample. To this end we will employ algorithms with controlled numerical error that can be reduced to any desired degree. The two methods that we choose are the time-evolving density matrix with orthogonal polynomials algorithm (TEDOPA)[5–7], to be described in this section, and a variation of the hierarchical equations of motion (HEOM) that we will describe in the following Supplementary Note 4.

The original formulation of TEDOPA for finite temperature environments requires the time consuming preparation of the thermal state of the environment and subsequent time evolution by means of matrix product operators (MPO)[8] as opposed to the zero temperature case for which the initial state of the environment is trivial and the system can be described by the more efficient matrix product state (MPS) formalism[5,6]. Recently, however, it was demonstrated that there is an exact analytical mapping of the finite temperature case to the zero temperature case which allows for the direct computation of system observables[7,9]. This approach, termed T-TEDOPA, leads to a major reduction in computational cost and, crucially, provides opportunities for further simplifications in the computations of absorption spectra that lead to additional significant reductions in computational cost by several orders of magnitude.

The calculation of the absorption spectrum requires the Fourier-Laplace transform of the correlation function $D(t)$ of the electronic transition dipole operator $\mu = \sum_i^N (\boldsymbol{\mu}_i \cdot \mathbf{e}) \left|\varepsilon_i\right\rangle \left\langle g\right| + h.c.$ averaged over orientational and energetic disorder[10] where $|\varepsilon_i\rangle$ denotes the local excitation at site i, $|g\rangle$ the global electronic ground state, $\boldsymbol{\mu}_i$ the dipole moment of site i and $\mathbf{e}$ the direction of polarisation of the electric field inducing absorption. This dipole-dipole correlation function is given by

$$\langle D(t)\rangle_{\mathbf{e},\varepsilon_i} = \langle \mathrm{Tr}[\mu e^{-iHt}\mu\rho_{th}e^{iHt}]\rangle_{\mathbf{e},\varepsilon_i}, \tag{13}$$

where $\rho_{th} = e^{-\beta H}/\mathrm{Tr}[e^{-\beta H}]$. Even well beyond physiological temperatures ρ_{th} is exceedingly well approximated by $\rho_{th} = |g\rangle\langle g| \otimes e^{-\beta H_v}/\mathrm{Tr}[e^{-\beta H_v}]$ as the exciton energy is typically found to be in the eV range. With this assumption, the T-TEDOPA approach obtains identically the same dynamics of electronic observables under a mapping that takes the initial state to $|g\rangle\langle g| \otimes |vac\rangle\langle vac|$, where $|vac\rangle$ denotes the vacuum state of the environment for which all vibrational modes are in their ground state and, simultaneously, takes the environmental spectral density from the original $J(\omega)$ on the interval $[0, \infty]$ to

$$J_\beta(\omega) = \frac{1}{2} sign(\omega) J(|\omega|) \left[1 + \coth\left(\frac{\beta\omega}{2}\right)\right], \tag{14}$$

which is now defined on the entire real axis $[-\infty, \infty]$. Then the dipole-dipole correlation function is reduced to

$$\langle D(t)\rangle_{\mathbf{e},\varepsilon_i} = \langle \mathrm{Tr}[\mu e^{-iH_\beta t}\mu \left|g\right\rangle\left\langle g\right| \otimes \left|vac\right\rangle\left\langle vac\right| e^{iH_\beta t}]\rangle_{\mathbf{e},\varepsilon_i}. \tag{15}$$

Now making use of $H|g\rangle|vac\rangle = 0$ and specialising to the case of a dimer ($N = 2$) we find

$$\begin{aligned}
\langle D(t)\rangle_{\mathbf{e},\varepsilon_i} &= \langle \mathrm{Tr}[\mu e^{-iH_\beta t}\mu \left|g\right\rangle\left\langle g\right| \otimes \left|vac\right\rangle\left\langle vac\right|]\rangle_{\mathbf{e},\varepsilon_i} && (16)\\
&= \langle \sum_{i,j=1}^{2} (\boldsymbol{\mu}_i \cdot \mathbf{e})^* (\boldsymbol{\mu}_j \cdot \mathbf{e}) \langle vac|\langle \varepsilon_i| e^{-iH_\beta t} |\varepsilon_j\rangle|vac\rangle\rangle_{\mathbf{e},\varepsilon_i} && (17)\\
&= \langle \sum_{i,j=1}^{2} (\boldsymbol{\mu}_i^* \cdot \boldsymbol{\mu}_j) \langle vac|\langle \varepsilon_i| e^{-iH_\beta t} |\varepsilon_j\rangle|vac\rangle\rangle_{\epsilon_i}. && (18)
\end{aligned}$$

The computational effort of TEDOPA simulations typically grows stronger than linear with the simulated time t, because it requires longer environmental chains and higher bond dimension of the employed matrix product states. Hence it proves advantageous to compute Eq. (18) via

$$|\psi_i(t/2)\rangle = e^{-iH_\beta t/2}|\varepsilon_i\rangle|vac\rangle = \left(e^{iH_\beta t/2}|\varepsilon_i\rangle|vac\rangle\right)^*,$$

so that

$$\langle D(t)\rangle_{\mathbf{e},\varepsilon_i} = \langle || \sum_{i=1}^{2} \boldsymbol{\mu}_j |\psi_j(t/2)\rangle||^2\rangle_{\epsilon_i}. \tag{19}$$

For the parameters in our work, this rearrangement of the dipole-dipole correlation function leads to more than one order of magnitude of reduction in the simulation time. The average over the energetic disorder can be simplified by

two measures. First, instead of an independent average over site energies ε_i, described by

$$\langle D(t)\rangle_{\mathbf{e},\varepsilon_i} = \frac{1}{2\pi\sigma^2}\int_{-\infty}^{\infty}\int_{-\infty}^{\infty} d\varepsilon_1 d\varepsilon_2 \exp\left(-\frac{1}{2\sigma^2}(\varepsilon_1-\langle\varepsilon_1\rangle)^2-\frac{1}{2\sigma^2}(\varepsilon_2-\langle\varepsilon_2\rangle)^2\right)||\sum_{i=1}^{2}\boldsymbol{\mu}_j|\psi_j(t/2)\rangle||^2, \tag{20}$$

we compute the ensemble average over $\xi_1=\varepsilon_1-\varepsilon_2$ and $\xi_2=\frac{1}{2}(\varepsilon_1+\varepsilon_2)$

$$\langle D(t)\rangle_{\mathbf{e},\varepsilon_i} = \frac{1}{2\pi\sigma^2}\int_{-\infty}^{\infty}\int_{-\infty}^{\infty} d\xi_1 d\xi_2 \exp\left(-\frac{1}{4\sigma^2}(\xi_1-\langle\xi_1\rangle)^2-\frac{1}{\sigma^2}(\xi_2-\langle\xi_2\rangle)^2\right)||\sum_{i=1}^{2}\boldsymbol{\mu}_j|\psi_j(t/2)\rangle||^2. \tag{21}$$

The ensemble average over $\xi_2=\frac{1}{2}(\varepsilon_1+\varepsilon_2)$ does not require the repetition of simulations, as the global shift in site energies does not affect electronic dynamics, but simply induces a time-dependent phase factor, which can be taken into account analytically. In other words, after numerical simulation of $||\sum_{i=1}^{2}\boldsymbol{\mu}_j|\psi_j(t/2)\rangle||^2_{\xi_1,\langle\xi_2\rangle}$ for $\xi_1=\varepsilon_1-\varepsilon_2$ and $\xi_2=\langle\xi_2\rangle=\frac{1}{2}(\langle\varepsilon_1\rangle+\langle\varepsilon_2\rangle)$, the average over ξ_2 can be computed by

$$\frac{1}{\sqrt{\pi}\sigma}\int_{-\infty}^{\infty} d\xi_2 e^{-i(\xi_2-\langle\xi_2\rangle)t_2}e^{-\frac{1}{\sigma^2}(\xi_2-\langle\xi_2\rangle)^2}||\sum_{i=1}^{2}\boldsymbol{\mu}_j|\psi_j(t/2)\rangle||^2_{\xi_1,\langle\xi_2\rangle} = \exp(-\frac{1}{4}\sigma^2t^2)||\sum_{i=1}^{2}\boldsymbol{\mu}_j|\psi_j(t/2)\rangle||^2_{\xi_1,\langle\xi_2\rangle}, \tag{22}$$

where the Gaussian dephasing $e^{-\frac{1}{4}\sigma^2t^2}$ leads to a broadening of absorption line shapes. The numerical average over energy difference $\xi_1=\varepsilon_1-\varepsilon_2$ can be carried out most efficiently by means of a Gauss-Hermite interpolation of the integral over the frequency difference. Compared to a straightforward averaging with equidistant sampling points, this leads to a reduction of computational effort by almost one order of magnitude.

A further significant step in the optimisation of the T-TEDOPA simulation concerns an essential part of the time-evolving block-decimation (TEBD) algorithm. This step selects the best MPS-approximation with fixed bond dimension through a decimation technique that relies on the singular value decomposition (SVD). As the complexity of each time-evolution step is dominated by the SVD we follow Refs. 11 and 12 and apply a randomized version of the SVD routine (rSVD) which trades a reduction in numerical accuracy for a significant increase in computational efficiency. At first sight, for the relatively moderate matrix sizes in our simulations, one may not expect significant computational gains due to the use of the rSVD. However, two specific aspects of the present problem lead to a different conclusion. As the absorption spectrum is obtained by the Fourier transform of the correlation function $D(t)$ and is further broadened due to the average over orientational and energetic disorder, small errors in the time evolution tend to be averaged out. Furthermore, the uncertainty in the experimentally determined environmental spectral density makes it unnecessary to aim for extremely high precision in the absorption spectra. A relative error of the order of about 10^{-3} suffices for our application. This allows us to accept a larger tolerated error in the rSVD which increases its speed significantly. As a result of the use of the rSVD we have been able to obtain more than one order of magnitude reduction in CPU time without noticeably affecting the quality of the results. As a result of these and some other minor measures, the computation of a single absorption spectrum fully averaged over energetic and orientational disorder for the full spectral density of WSCP takes about 3 minutes on 7 processors (2 GHz).

We note that the simulation cost of TEDOPA can be reduced further by transforming the two local baths to two effective baths describing the center-of-mass and the relative motion of the local modes with identical vibrational frequencies (see Supplementary Note 4). As the influence of the center-of-mass modes on the reduced electronic system dynamics can be treated analytically, one can remove the center-of-mass modes from the dynamics and hence the TEDOPA simulations and consider a dimeric electronic system coupled to a single global bath describing the relative motion modes. The application of orthogonal polynomial algorithm[5–7] to this model leads to a single chain of coupled harmonic oscillators where the first oscillator is also coupled to the electronic states of a dimeric system. This enables one to reduce the simulation cost of TEDOPA compared to the case that two local baths are mapped to two chains coupled via the electronic states of the dimer. This also implies that one can consider a trimeric electronic system coupled to three local baths in TEDOPA simulations, as three local baths can be mapped to three global baths, including a bath describing the center-of-mass motions which can be neglected in simulations. In this case, the remaining two global baths, describing relative motion modes, can be mapped to two chains coupled via the electronic states of a trimer, which can be efficiently simulated within the framework of TEDOPA.

Supplementary Note 4. HEOM

Hierarchical equations of motion (HEOM) are a numerically exact method for computing reduced system dynamics where a quantum system is linearly coupled to a thermal environment modelled by quantum harmonic oscillators. In

the following we will explain the specifics of our HEOM implementation including the measures that we have taken to optimise the calculation of absorption spectra.

We assume that at the initial time, system and environment are uncorrelated and the environment is in a thermal state at temperature T. In this work, we consider a local bath model where each site is coupled to an independent bath modelled by an identical phonon spectral density $J(\omega)$, characterizing system-environment coupling strengths as a function of environmental mode frequencies ω. The influence of such a harmonic bath on the reduced system dynamics is fully characterized by the bath correlation function

$$C(t) = \int_0^\infty d\omega J(\omega) \left(\coth\left(\frac{\beta\omega}{2}\right) \cos(\omega t) - i \sin(\omega t) \right). \tag{23}$$

The parameters of HEOM simulations are determined by a multi-exponential fitting of the bath correlation function

$$C(t) \approx \sum_{j=1}^{N_C} (d_{j+} e^{-f_j t} + d_{j-} e^{-f_j^* t}) + \sum_{k=1}^{N_R} d_k e^{-\gamma_k t}, \tag{24}$$

where the fitting variables d_{j+}, d_{j-}, f_j, d_k are complex-valued, while γ_k are real-valued. The total number of exponential functions is given by $2N_C + N_R$, which is one of the key factors determining the simulation cost of HEOM. The simulation of reduced system dynamics within a finite time window, $0 \leq t \leq t_{\max}$, requires the multi-exponential fitting of $C(t)$ within that time window, as the fitting quality outside of the time window of interest, namely $t > t_{\max}$, does not affect the system dynamics for $0 \leq t \leq t_{\max}$. The fitting quality can be monitored by comparing simulated HEOM results with the analytical solutions of solvable models or the data obtained by alternative numerically exact methods. In this work, we consider an analytically solvable monomer model, namely a two-level system under dephasing noise. For a given set of fitting parameters, the dynamics of the reduced system density matrix $\hat{\rho}_{\mathbf{0}}$ can be non-perturbatively computed by using HEOM in the form

$$\begin{aligned}
\frac{d\hat{\rho}_{\mathbf{n}}}{dt} = &-\frac{i}{\hbar}[\hat{H}_s, \hat{\rho}_{\mathbf{n}}] - \sum_{m=1}^{N_S} \left(\sum_{j=1}^{N_C} (n_{mj+} f_j + n_{mj-} f_j^*) + \sum_{k=1}^{N_R} n_{mk}\gamma_k \right) \hat{\rho}_{\mathbf{n}} - i \sum_{m=1}^{N_S} \left[\hat{\sigma}_m^\dagger \hat{\sigma}_m, \sum_{j=1}^{N_C} (\hat{\rho}_{\mathbf{n}_{mj+}^+} + \hat{\rho}_{\mathbf{n}_{mj-}^+}) + \sum_{k=1}^{N_R} \hat{\rho}_{\mathbf{n}_{mk}^+} \right] \\
&- i \sum_{m=1}^{N_S} \sum_{j=1}^{N_C} n_{mj+} \left(d_{j+} \hat{\sigma}_m^\dagger \hat{\sigma}_m \hat{\rho}_{\mathbf{n}_{mj+}^-} - d_{j-}^* \hat{\rho}_{\mathbf{n}_{mj+}^-} \hat{\sigma}_m^\dagger \hat{\sigma}_m \right) - i \sum_{m=1}^{N_S} \sum_{j=1}^{N_C} n_{mj-} \left(d_{j-} \hat{\sigma}_m^\dagger \hat{\sigma}_m \hat{\rho}_{\mathbf{n}_{mj-}^-} - d_{j+}^* \hat{\rho}_{\mathbf{n}_{mj-}^-} \hat{\sigma}_m^\dagger \hat{\sigma}_m \right) \\
&- i \sum_{m=1}^{N_S} \sum_{k=1}^{N_R} n_{mk} \left(d_k \hat{\sigma}_m^\dagger \hat{\sigma}_m \hat{\rho}_{\mathbf{n}_{mk}^-} - d_k^* \hat{\rho}_{\mathbf{n}_{mk}^-} \hat{\sigma}_m^\dagger \hat{\sigma}_m \right),
\end{aligned} \tag{25}$$

where N_S denotes the number of sites of the system ($N_S = 1$ for a monomer, $N_S = 2$ for a dimer, such as WSCP and the special pair in bacterial reaction centers), and $\hat{\sigma}_m$ ($\hat{\sigma}_m^\dagger$) is the annihilation (creation) operator of an electronic excitation at site m. The information about the reduced system dynamics and system-environment correlations is included in the auxiliary operators $\hat{\rho}_{\mathbf{n}}$, which are labelled by vectors $\mathbf{n} = (n_{mj+}, \cdots, n_{mj-}, \cdots, n_{mk}, \cdots)$ with non-negative integer elements. The sum of the integer elements of the vector $\mathbf{n}$ is denoted by N_{rank}. The reduced system density matrix is described by the lowest-rank operator $\hat{\rho}_{\mathbf{0}}$ with $N_{\text{rank}} = 0$ where all the integer elements of $\mathbf{n}$ are zero. All the other higher-rank auxiliary operators contain the information about system-environment correlations and have the same dimension as the reduced system density matrix $\hat{\rho}_{\mathbf{0}}$ and are taken to be null matrices at the initial time. The interaction between auxiliary operators is described by $\mathbf{n}_{mj+}^\pm$, $\mathbf{n}_{mj-}^\pm$, $\mathbf{n}_{mk}^\pm$, which are defined by $\mathbf{n}_{mj+}^\pm = (n_{mj+} \pm 1, \cdots, n_{mj-}, \cdots, n_{mk}, \cdots)$, $\mathbf{n}_{mj-}^\pm = (n_{mj+}, \cdots, n_{mj-} \pm 1, \cdots, n_{mk}, \cdots)$, $\mathbf{n}_{mk}^\pm = (n_{mj+}, \cdots, n_{mj-}, \cdots, n_{mk} \pm 1, \cdots)$, which are one-rank higher or lower than $\mathbf{n}$. To obtain numerically exact results, one needs to increase the number of auxiliary operators, determined by the maximum value of N_{rank}, until the simulated system dynamics shows numerical convergence. In this work, the convergence of HEOM simulations is achieved for the maximum $N_{\text{rank}} = 10$.

In absorption simulations, we compute the dynamics of optical coherence in the form $\sum_{i=1}^{N_S} \psi_i(t)|\varepsilon_i\rangle\langle g|$, where $|\varepsilon_i\rangle$ denotes a singly excited state of site i, and $|g\rangle$ is the global electronic ground state. Note that $\hat{\sigma}_m^\dagger \hat{\sigma}_m |g\rangle = 0$ due to the absence of electronic excitations in the global ground state. This implies that in absorption simulations, the mathematical form of HEOM can be simplified to

$$\begin{aligned}
\frac{d\hat{\rho}_{\mathbf{n}}}{dt} = &-\frac{i}{\hbar}\hat{H}_s \hat{\rho}_{\mathbf{n}} - \sum_{m=1}^{N_S} \left(\sum_{j=1}^{N_C} (n_{mj+} f_j + n_{mj-} f_j^*) + \sum_{k=1}^{N_R} n_{mk}\gamma_k \right) \hat{\rho}_{\mathbf{n}} - i \sum_{m=1}^{N_S} \hat{\sigma}_m^\dagger \hat{\sigma}_m \left(\sum_{j=1}^{N_C} (\hat{\rho}_{\mathbf{n}_{mj+}^+} + \hat{\rho}_{\mathbf{n}_{mj-}^+}) + \sum_{k=1}^{N_R} \hat{\rho}_{\mathbf{n}_{mk}^+} \right) \\
&- i \sum_{m=1}^{N_S} \sum_{j=1}^{N_C} n_{mj+} d_{j+} \hat{\sigma}_m^\dagger \hat{\sigma}_m \hat{\rho}_{\mathbf{n}_{mj+}^-} - i \sum_{m=1}^{N_S} \sum_{j=1}^{N_C} n_{mj-} d_{j-} \hat{\sigma}_m^\dagger \hat{\sigma}_m \hat{\rho}_{\mathbf{n}_{mj-}^-} - i \sum_{m=1}^{N_S} \sum_{k=1}^{N_R} n_{mk} d_k \hat{\sigma}_m^\dagger \hat{\sigma}_m \hat{\rho}_{\mathbf{n}_{mk}^-},
\end{aligned} \tag{26}$$

where the auxiliary operators are described by $\hat{\rho}_{\mathbf{n}} = \sum_{i=1}^{N_S} \psi_{\mathbf{n},i}(t)|\varepsilon_i\rangle\langle g|$, similar to N_S-dimensional states with unnormalized amplitudes $\psi_{\mathbf{n},i}(t)$. We note that $\hat{H}_s$ is the electronic Hamiltonian in this work, satisfying $\hat{H}_s|g\rangle = 0$. It is notable that in Eq. (25), d_{j+}, namely the amplitude of $e^{-f_j t}$ considered in the multi-exponential fitting (see Eq. (24)), induces the coupling between $\hat{\rho}_{\mathbf{n}}$ and $\hat{\rho}_{\mathbf{n}^-_{mj+}}$ (see d_{j+} in Eq. (25)) and that between $\hat{\rho}_{\mathbf{n}}$ and $\hat{\rho}_{\mathbf{n}^-_{mj-}}$ (see d^*_{j+} in Eq. (25)). This implies that even if we consider only $d_{j+}e^{-f_j t}$ in Eq. (24) by setting $d_{j-} = 0$, the auxiliary operators are labelled by $\mathbf{n} = (n_{mj+}, \cdots, n_{mj-}, \cdots, n_{mk}, \cdots)$ with both n_{mj+} and n_{mj-} included, as is the case that both $d_{j+}e^{-f_j t}$ and $d_{j-}e^{-f^*_j t}$ are considered in the fitting. Hence, setting $d_{j-} = 0$ does not reduce the number of auxiliary operators for a given maximum value of N_{rank} in general. However, in absorption simulations, as shown in Eq. (26), d_{j+} only induces the interaction between $\hat{\rho}_{\mathbf{n}}$ and $\hat{\rho}_{\mathbf{n}^-_{mj+}}$, not the interaction between $\hat{\rho}_{\mathbf{n}}$ and $\hat{\rho}_{\mathbf{n}^-_{mj-}}$. Therefore, in absorption simulations, one does not need to fit the bath correlation function with pairwise exponentials, $d_{j+}e^{-f_j t} + d_{j-}e^{-f^*_j t}$, and can consider a sum of fully independent exponentials in the form

$$C(t) \approx \sum_{j=1}^{N_C} d_j e^{-f_j t}, \tag{27}$$

with complex-valued d_j and f_j. In this case, HEOM is simplified to

$$\frac{d\hat{\rho}_{\mathbf{n}}}{dt} = -\frac{i}{\hbar}\hat{H}_s\hat{\rho}_{\mathbf{n}} - \sum_{m=1}^{N_S}\sum_{j=1}^{N_C} n_{mj} f_j \hat{\rho}_{\mathbf{n}} - i\sum_{m=1}^{N_S} \hat{\sigma}^\dagger_m \hat{\sigma}_m \sum_{j=1}^{N_C} \hat{\rho}_{\mathbf{n}^+_{mj}} - i\sum_{m=1}^{N_S}\sum_{j=1}^{N_C} n_{mj} d_j \hat{\sigma}^\dagger_m \hat{\sigma}_m \hat{\rho}_{\mathbf{n}^-_{mj}}, \tag{28}$$

where $\mathbf{n} = (n_{mj}, \cdots)$. This approach provides more flexibility in the multi-exponential fitting of the bath correlation function and can reduce the number of exponentials required to achieve a desired fitting quality. We note that this approach cannot be employed when the population dynamics of electronic excited states is involved in the physical quantity of interest, such as nonlinear optical responses.

To further reduce the simulation cost of HEOM, we map local vibrational modes to global modes including the center-of-mass modes. This enables one to perform HEOM simulations without considering the center-of-mass modes, which reduces the dimension of the vectors $\mathbf{n}$ labelling auxiliary operators $\hat{\rho}_{\mathbf{n}}$. As an example, we consider a dimer model where each site is coupled to a single local mode with an identical vibrational frequency ω and a Huang-Rhys factor s

$$H = H_e + \sum_{i=1}^{2} \left(\omega b^\dagger_i b_i + \omega\sqrt{s}|\varepsilon_i\rangle\langle\varepsilon_i| \otimes (b^\dagger_i + b_i)\right). \tag{29}$$

By introducing a center-of-mass mode $B = \frac{1}{\sqrt{2}}(b_1 + b_2)$ and a relative-motion mode $b = \frac{1}{\sqrt{2}}(b_1 - b_2)$, satisfying the bosonic commutation relations, the Hamiltonian can be expressed as

$$H = H_e + \omega(B^\dagger B + b^\dagger b) + \omega\sqrt{s}A_+ \otimes (B^\dagger + B) + \omega\sqrt{s}A_- \otimes (b^\dagger + b), \tag{30}$$

where $A_\pm = (|\varepsilon_1\rangle\langle\varepsilon_1| \pm |\varepsilon_2\rangle\langle\varepsilon_2|)/\sqrt{2}$. It is notable that $A_+ \propto |\varepsilon_1\rangle\langle\varepsilon_1| + |\varepsilon_2\rangle\langle\varepsilon_2|$ is proportional to the identity operator of the single electronic excitation subspace. This implies that the center-of-mass mode induces fully correlated fluctuations of the energy levels of the two sites, without modifying the energy-level difference between them. Therefore the influence of the center-of-mass mode on the dynamics of optical coherence can be computed in an analytical way, as is the case of the Kubo's lineshape theory for a two-level monomer. The influence of the relative-motion mode on the system dynamics, described by A_-, still requires numerically exact simulations. The mapping from local to global modes can be generalized to a multi-mode case characterized by a phonon spectral density. For a dimer system coupled to identical local baths, the influence of the relative-motion modes on optical coherence can be computed by HEOM in the form

$$\frac{d\hat{\rho}_{\mathbf{n}}}{dt} = -\frac{i}{\hbar}\hat{H}_s\hat{\rho}_{\mathbf{n}} - \sum_{j=1}^{N_C} n_j f_j \hat{\rho}_{\mathbf{n}} - i\frac{|\varepsilon_1\rangle\langle\varepsilon_1| - |\varepsilon_2\rangle\langle\varepsilon_2|}{\sqrt{2}} \sum_{j=1}^{N_C} \hat{\rho}_{\mathbf{n}^+_j} - i\sum_{j=1}^{N_C} n_j d_j \frac{|\varepsilon_1\rangle\langle\varepsilon_1| - |\varepsilon_2\rangle\langle\varepsilon_2|}{\sqrt{2}} \hat{\rho}_{\mathbf{n}^-_j}, \tag{31}$$

where $\mathbf{n} = (n_j, \cdots)$. It is notable that the dimension of $\mathbf{n} = (n_j, \cdots)$ of the relative-motion model is the half of the dimension of $\mathbf{n} = (n_{1j}, \cdots, n_{2j}, \cdots)$ of the original model with two local baths. This significantly reduces the number of auxiliary operators for a given maximum value of N_{rank} and corresponding simulation cost. This approach can be generalized to a multi-site system consisting of N_S sites where $\mathbf{n} = (n_{1j}, \cdots, n_{N_S j}, \cdots)$ is reduced to $\mathbf{n} = (n_{1j}, \cdots, n_{N_S-1,j}, \cdots)$ by removing a center-of-mass bath.

We note that the dynamics of the optical coherence $\hat{\rho}_{\mathbf{0}}(t) = \sum_{i=1}^{2} \psi_{\mathbf{0},i}(t)|\varepsilon_i\rangle\langle g|$ computed by Eq. (31) only considers the dephasing induced by the relative-motion modes. The additional dephasing caused by the center-of-mass modes can be taken into account analytically by multiplying an additional time-dependent factor $\exp(-\frac{1}{2}G(t))$ that is independent of the electronic excited states $|\varepsilon_i\rangle$, namely $\sum_{i=1}^{2} \psi_{\mathbf{0},i}(t)|\varepsilon_i\rangle\langle g| \exp(-\frac{1}{2}G(t))$ where

$$G(t) = -i\lambda t + \int_0^\infty d\omega \frac{J(\omega)}{\omega^2} \left((1 - \cos(\omega t)) \left(1 + \frac{2}{e^{\omega/k_B T} - 1} \right) + i \sin(\omega t) \right), \tag{32}$$

with the reorganization energy $\lambda = \int_0^\infty d\omega J(\omega)/\omega$. The factor 1/2 in $\exp(-\frac{1}{2}G(t))$, which does not appear in the conventional lineshape theory, originates from the $1/\sqrt{2}$ factor in A_+ describing the coupling to the center-of-mass modes. We note that this approach can be generalized to the simulations of nonlinear optical spectra by taking into account the contribution of the center-of-mass modes analytically.

We would like to stress that the techniques devised to reduce the computational cost of absorption simulations by T-TEDOPA can also be applied to HEOM. As explained in Supplementary Note 3, in T-TEDOPA the finite temperature environments can be mapped to zero temperature environments for which the initial state becomes a pure state, while the phonon spectral density becomes temperature-dependent. This enables one to compute the dipole-dipole correlation function up to time t by simulating the time evolution of a pure electronic-vibrational state up to time $t/2$. This technique can be employed in HEOM. In absorption simulations, the initial state of the system density matrix is given by $\hat{\rho}_{\mathbf{0}} = |g\rangle\langle g|$ and all the higher-rank auxiliary operators are taken to be null matrices at the initial time, namely $\hat{\rho}_{\mathbf{n}} = 0$ for all $\mathbf{n} \neq \mathbf{0}$. When a transition dipole moment operator μ is multiplied to the left-hand side of the auxiliary operators, the lowest-rank operator becomes a linear combination of optical coherences, $\hat{\rho}_{\mathbf{0}}(0) = \sum_{i=1}^{N_S} \psi_{\mathbf{0},i}(0)\,|\varepsilon_i\rangle\langle g|$. In this case, as shown in Eq. (28), the time evolution of the auxiliary operators is governed by a propagator that is multiplied only to the left-hand side of $\hat{\rho}_{\mathbf{n}}$. This makes it straightforward to define a high-dimensional vector $\boldsymbol{\rho}(t)$, consisting of all the elements $\psi_{\mathbf{n},i}(t)$ of the auxiliary operators $\hat{\rho}_{\mathbf{n}}(t) = \sum_{i=1}^{N_S} \psi_{\mathbf{n},i}(t)\,|\varepsilon_i\rangle\langle g|$, whose dynamics is governed by $\frac{d}{dt}\boldsymbol{\rho}(t) = G\boldsymbol{\rho}(t)$ with a propagator G. The dipole-dipole correlation function is then described by

$$\langle D(t)\rangle_{\mathbf{e},\varepsilon_i} = \langle \boldsymbol{\rho}_{\mathbf{n}}(0) \cdot (e^{Gt}\boldsymbol{\rho}_{\mathbf{n}}(0))\rangle_{\mathbf{e},\varepsilon_i} = \langle (e^{G^\dagger t/2}\boldsymbol{\rho}_{\mathbf{n}}(0)) \cdot (e^{Gt/2}\boldsymbol{\rho}_{\mathbf{n}}(0))\rangle_{\mathbf{e},\varepsilon_i}, \tag{33}$$

demonstrating that one can reduce the simulation time from t to $t/2$ in HEOM simulations, as is the case of T-TEDOPA. The reduced time window can decrease the number of auxiliary operators required to obtain numerically exact absorption line shapes, as higher-rank auxiliary operators are initially null matrices and they are populated only by the interaction with lower-rank auxiliary operators.

The computational cost of HEOM is determined by the number of the auxiliary operators, controlled by the maximum value of N_{rank}. As detailed in Supplementary Note 6, we consider experimentally estimated phonon spectral densities, including 55 intra-pigment modes per site, and perform HEOM simulations with the maximum $N_{\text{rank}} = 10$ to obtain fully converged absorption spectra for the cases that the bath correlation functions of WSCP and SP are fitted with the sum of 13 and 16 exponentials, respectively. For the maximum $N_{\text{rank}} = 10$ and $N_{\text{exp}} = 13$ (or $N_{\text{exp}} = 16$) exponentials, the number of the auxiliary operators is given by $(N_{\text{exp}} + N_{\text{rank}})!/(N_{\text{exp}}!N_{\text{rank}}!)$. For absorption simulations of a dimeric system, it is sufficient to consider a subspace spanned by optical coherences $|\varepsilon_1\rangle\langle g|$ and $|\varepsilon_2\rangle\langle g|$ for each auxiliary operator, leading to the simulation cost of 37 MB and 170 MB, respectively, for WSCP and SP.

When the experimentally estimated spectral densities are fitted with Drude-Lorentz peaks, one needs to consider at least two exponentials to describe each local intra-pigment mode. In such a conventional HEOM approach, a dimeric system coupled to 55 intra-pigment modes per site corresponds to a case of 220 exponentials. In this case, the simulation cost for the maximum $N_{\text{rank}} = 10$ becomes of the order of 10^6 TB. Even if the number of auxiliary operators is reduced for approximate HEOM simulations, the simulation cost is of the order of 1 TB for the maximum $N_{\text{rank}} = 6$, for which we found that the numerical errors of simulated absorption spectra are not negligible. We note that this estimate does not include the number of exponentials required to consider low-frequency protein motions and Matsubara frequencies originating from the intra-pigment modes, implying that the computational cost of the conventional approach can be even higher than the estimate provided here.

Supplementary Note 5. Electronic Parameters and Phonon Spectral Densities of WSCP and SP

Here we provide a summary of the electronic parameters and phonon spectral densities of WSCP and SP considered in simulations.

The electronic parameters of WSCP have been estimated based on the phonon spectral density of B777 photosynthetic complexes[13]

$$J_l^{\mathrm{B777}}(\omega) = \frac{S}{s_1+s_2}\sum_{i=1}^{2}\frac{s_i}{7!2\omega_i^4}\omega^5 e^{-(\omega/\omega_i)^{1/2}}, \tag{34}$$

where $S = 0.8$, $s_1 = 0.8$, $s_2 = 0.5$, $\omega_1 = 0.069\,\mathrm{meV}$ and $\omega_2 = 0.24\,\mathrm{meV}$. By using second order cumulant expansion, it is found that the low-energy part of experimental absorption spectra of WSCP can be well reproduced when the electronic coupling between two pigments is $V = 69\,\mathrm{cm}^{-1}$ and the angle between monomer transition dipole moments is 39°.

The phonon spectral density of WSCP has been estimated based on experimentally measured fluorescence line-narrowing spectra of WSCP[1,14]. It is found that the low-frequency part of the phonon spectral density can be well described by a sum of three log-normal distributions in the form

$$J_l^{\mathrm{WSCP}}(\omega) = \sum_{m=1}^{3}\frac{S_m}{\sigma_m\sqrt{2\pi}}\omega\exp(-\frac{[\ln(\omega/\Omega_m)]^2}{2\sigma_m^2}), \tag{35}$$

with $S_1 = 0.39$, $S_2 = 0.23$, $S_3 = 0.23$, $\sigma_1 = 0.4$, $\sigma_2 = 0.25$, $\sigma_3 = 0.2$, $\Omega_1 = 26\,\mathrm{cm}^{-1}$, $\Omega_2 = 51\,\mathrm{cm}^{-1}$, $\Omega_3 = 85\,\mathrm{cm}^{-1}$. The high-frequency part of the phonon spectral density consists of 55 intra-pigment vibrational modes, which are modelled by Lorentzian functions in the form

$$J_h(\omega) = \sum_{k=1}^{55}\frac{4\omega_k s_k\gamma_k(\omega_k^2+\gamma_k^2)\omega}{\pi((\omega+\omega_k)^2+\gamma_k^2)((\omega-\omega_k)^2+\gamma_k^2)}, \tag{36}$$

where vibrational frequencies ω_k and Huang-Rhys factors s_k are summarised in Supplementary Table 1. The vibrational damping rates of the intra-pigment modes are taken to be $\gamma_k = (1\,\mathrm{ps})^{-1}$. It is found that experimentally measured absorption spectra of WSCP can be reproduced by numerically exact simulations when $J_l^{\mathrm{WSCP}}(\omega) + J_h(\omega)$ and $V = 140\,\mathrm{cm}^{-1}$ are considered with independent static disorder in site energies modelled by Gaussian distributions with the standard deviation of $80\,\mathrm{cm}^{-1}$ (see Supplementary Note 6).

The electronic parameters of SP heterodimers have been estimated based on experimentally measured absorption, linear dichroism and hole burning spectra of bacterial reaction centers[15]. By using conventional line shape theory, it is found that the difference in mean site energies is $\langle\varepsilon_1 - \varepsilon_2\rangle = 315\,\mathrm{cm}^{-1}$, electronic coupling is $V = 625\,\mathrm{cm}^{-1}$, and the angle between transition dipole moments of monomers is 143°.

Experimentally estimated phonon spectral density of SP[2,16] consists of a log-normal distribution function

$$J_l^{\mathrm{SP}}(\omega) = \frac{S_l}{\sigma_l\sqrt{2\pi}}\omega\exp(-\frac{[\ln(\omega/\Omega_l)]^2}{2\sigma_l^2}), \tag{37}$$

with $S_l = 1.7$, $\sigma_l = 0.47$, $\Omega_l = 35\,\mathrm{cm}^{-1}$, and the special pair marker mode with vibrational frequency $\omega_{\mathrm{sp}} = 125\,\mathrm{cm}^{-1}$, Huang-Rhys factor $s_{\mathrm{sp}} = 1.5$ and damping rate $\gamma_{\mathrm{sp}} = 15\,\mathrm{cm}^{-1}$, and 55 intra-pigment vibrational modes of BChl$a$ pigments with vibrational frequencies and Huang-Rhys factors summarised in Supplementary Table 2. The damping rates of the intra-pigment modes are taken to be $\gamma_k = (1\,\mathrm{ps})^{-1}$. The special pair marker mode and 55 intra-pigment modes are modelled by Lorentzian spectral densities. In experiments where phonon spectral densities are estimated, the optical response may originate from the lowest-energy exciton rather than the lowest-energy pigment. To take into account this effect approximately, we consider diagonal vibronic couplings of the lowest-energy exciton state of bacterial reaction centers, namely the low-energy exciton $|E_-\rangle$ of the SP, where $\langle E_-|\,H_{e-v}\,|E_-\rangle = \omega\sqrt{s}(\sin^2(\theta/2)(b_1 + b_1^\dagger) + \cos^2(\theta/2)(b_2 + b_2^\dagger))$. We introduce an effective mode $b = (\sin^4(\theta/2) + \cos^4(\theta/2))^{-1/2}(\sin^2(\theta/2)b_1 + \cos^2(\theta/2)b_2)$, satisfying the bosonic commutation relation $[b, b^\dagger] = 1$, so that the diagonal vibronic couplings are expressed as $\langle E_-|\,H_{e-v}\,|E_-\rangle = \omega\sqrt{s_{E_-}}(b + b^\dagger)$ where the renormalised Huang-Rhys factor of the exciton state is given by $s_{E_-} = s(\sin^4(\theta/2) + \cos^4(\theta/2))$. Since experimental data are fitted with line shape functions based on the effective Huang-Rhys factor s_{E_-} in the estimation of phonon spectral densities, we renormalise the Huang-Rhys factors of the local modes, $s = s_{E_-}/(\sin^4(\theta/2) + \cos^4(\theta/2)) \approx 1.9\,s_{E_-}$ based on the mean site energy difference $\langle\varepsilon_1 - \varepsilon_2\rangle = 315\,\mathrm{cm}^{-1}$ and electronic coupling $V = 625\,\mathrm{cm}^{-1}$ of the SP. Since the Huang-Rhys factors S_l and s_{sp} of the log-normal distribution and special pair marker mode have been estimated in experiments on excitonic systems[16], we multiply the renormalisation factor ~ 1.9 to S_l and s_{sp}. Since the parameters in Supplementary Table 2 have been estimated in experiments on BChl$a$ monomers[2], we do not renormalise the Huang-Rhys factors s_k of the intra-pigment modes. It is found that the low-energy part of experimental absorption spectra of bacterial reaction centers, dominated by the optical responses of SP, can be well reproduced by numerically exact simulations when the experimentally estimated spectral density is considered with local static disorder modelled by Gaussian distributions with the standard deviation of $105\,\mathrm{cm}^{-1}$ (see Supplementary Note 6).

Supplementary Note 6. Numerical Test and Comparison of HEOM and TEDOPA for Structured Environments at Low Temperatures

In this section we provide direct evidence that both HEOM and TEDOPA are capable of achieving numerically exact results for dimeric systems in contact with realistic, highly structured environmental spectral densities. To this end we apply both methods to WSCP and the special pair in bacterial reaction centers and show that the fully converged results coincide.

Supplementary Figure 1 presents the HEOM and TEDOPA results of numerically exact absorption line shapes of WSCP at 77 K. Supplementary Figure 1a shows the experimentally estimated spectral density of WSCP which consists of three log-normal functions at low vibrational frequencies and 55 narrow Lorentzian functions corresponding to intra-pigment vibrational modes (see Supplementary Note 5 for all the parameters that enter this spectral density). For the HEOM simulation, the real and imaginary part of the corresponding bath correlation function at $T = 77\,\mathrm{K}$ is fitted with the sum of 13 exponentials, as shown in Supplementary Figure 1b. The quality of the fit is confirmed by its ability to reproduce the analytical solution of monomer optical coherence dynamics, as shown in Supplementary Figure 1c. Finally, Supplementary Figure 1d shows numerically exact absorption line shapes of WSCP obtained by independent calculations using HEOM and TEDOPA. For two different electronic coupling strengths $V = 69\,\mathrm{cm}^{-1}$ and $V = 140\,\mathrm{cm}^{-1}$, we observe perfect overlap of the absorption spectra obtained by the two methods. This demonstrates the reliability of the two methods and our simulated data.

Supplementary Figure 2 presents HEOM and TEDOPA results of numerically exact absorption line shapes of the special pair in bacterial reaction centers at $T = 5\,\mathrm{K}$ under the highly structured environmental spectral density shown in Supplementary Figure 2a. The corresponding bath correlation function at $T = 5\,\mathrm{K}$, shown in black in Supplementary Figure 2b, is well fitted with the sum of 16 exponentials up to $t = 200\,\mathrm{fs}$, as shown in red. Supplementary Figure 2c shows that the fitting quality is good enough to reproduce the analytical solution of monomer optical coherence dynamics. Supplementary Figure 2d displays the absorption spectra of the special pair demonstrating that the results obtained by HEOM and TEDOPA are well matched. Our results demonstrate that low-temperature systems can be efficiently simulated by HEOM when the bath correlation function is numerically fitted with exponentials. This contrasts the conventional approach where the bath correlation function is expanded as a sum of exponentials in an analytical way for some model spectral densities. A well-known example is the Ohmic spectral density with the Lorentz-Drude cutoff function where the number of exponentials required to achieve a desired fitting quality increases as temperature decreases, due to Matsubara terms, making HEOM simulations more challenging at lower temperatures. Even in this case, the simulation cost can be significantly reduced by replacing multiple Matsubara (exponential) terms with a few damped oscillations based on numerical fitting.

It is notable that the input parameters of HEOM simulations are determined based on the fitting of the bath correlation functions. This implies that one needs to improve the fitting quality until simulated HEOM results show convergence, which requires the repetition of HEOM simulations and as a result increases the overall simulation cost. This is contrary to TEDOPA where the input parameters are computed by orthogonal polynomial algorithm, which makes it easier to control the accuracy of input parameters when compared to HEOM.

Supplementary Note 7. Reduced Vibronic Model for Absorption Spectra

Numerically exact absorption line shape of the special pair in bacterial reaction centers consists of two narrow peaks centered at 780 and 900 nm, respectively, and a relatively broad peak around 800-850 nm. Here we show that the 780 nm and 900 nm peaks originate from vibronic eigenstates $|\psi_+\rangle$ and $|\psi_-\rangle$, resulting from the vibronic mixing of exciton states $|E_+\rangle$ and $|E_-\rangle$ with multiple intra-pigment modes, and the other vibrationally excited eigenstates contribute to the vibrational sideband in the 800-850 nm region.

To understand the origin of the three-peak structure, we consider the vibronic Hamiltonian where the excitons are coupled to the relative motions of 55 intra-pigment modes (see Supplementary Note 1)

$$H \approx H_e + \sum_{k=1}^{55} \omega_k b_k^\dagger b_k + \sum_{i,j} A_{ij} |E_i\rangle\langle E_j| \sum_{k=1}^{55} \omega_k \sqrt{s_k/2}(b_k^\dagger + b_k).$$

For simplicity, the relative motions of low-frequency protein modes and the vibrational damping of the relative intra-pigment modes b_k are not considered, which can induce pure dephasing and relaxation of vibronic eigenstates (see Supplementary Note 8). The influence of the center-of-mass motions of the protein and intra-pigment modes on absorption line shapes is treated non-perturbatively. In more detail, absorption line shape is determined by the Fourier transformation of the dynamics of optical coherence, $\mathrm{Tr}\{\mu e^{-iHt} \mu \rho_{th} e^{iHt}\} \exp(-\frac{1}{2}G(t))$ where $e^{\pm iHt}$ describes

the interaction between excitons and relative intra-pigment modes, while $G(t)$ takes into account the frequency shift and dephasing of the optical coherence induced by the center-of-mass motions

$$G(t) = \int_0^\infty d\omega \frac{J(\omega)}{\omega^2}[(1-\cos(\omega t))(1+\frac{2}{e^{\omega/k_BT}-1}) + i\sin(\omega t)] - i\lambda t, \quad (38)$$

where $J(\omega)$ denotes experimentally estimated phonon spectral density of SP, and $\lambda = \int_0^\infty d\omega J(\omega)/\omega$ is the reorganisation energy. To reduce the total number of vibrational excitations of the relative motion modes b_k required to obtain numerically converged absorption spectra, we consider the polaron transformation in Supplementary Note 1 where the vibronic Hamiltonian is transformed to

$$\tilde{H} = H_e + \sum_{k=1}^{55}\omega_k b_k^\dagger b_k - \sum_{i=\pm}|E_i\rangle\langle E_i|\left(\sum_{k=1}^{55}\omega_k\frac{s_k}{2}A_{ii}^2\right) \quad (39)$$

$$+ |E_+\rangle\langle E_-|A_{+-}\sum_{k=1}^{55}\omega_k\sqrt{\frac{s_k}{2}}D_k\left(\sqrt{\frac{s_k}{2}}A_{++}\right)(b_k^\dagger + b_k)D_k^\dagger\left(\sqrt{\frac{s_k}{2}}A_{--}\right)\prod_{k'\neq k}D_{k'}\left(\sqrt{\frac{s_{k'}}{2}}(A_{++}-A_{--})\right) + h.c.$$

Similarly the transition dipole moment operator is transformed to

$$\tilde{\mu} = (\mathbf{e}\cdot\boldsymbol{\mu}_{E_+})|E_+\rangle\langle g|\prod_{k=1}^{55}D_k\left(\sqrt{\frac{s_k}{2}}A_{++}\right) + (\mathbf{e}\cdot\boldsymbol{\mu}_{E_-})|E_-\rangle\langle g|\prod_{k=1}^{55}D_k\left(\sqrt{\frac{s_k}{2}}A_{--}\right) + h.c., \quad (40)$$

where $\boldsymbol{\mu}_{E_j} = \sum_{i=1}^2 \boldsymbol{\mu}_i\langle E_j|\varepsilon_i\rangle$ represent the transition dipole moment vectors of the exciton states $|E_+\rangle$ and $|E_-\rangle$ with $\boldsymbol{\mu}_{1,2}$ denoting the transition dipole moment vectors of monomers, and $\mathbf{e}$ is a unit vector describing polarization of the electric field inducing absorption.

For isotropic samples, one needs to consider the orientational average of absorption line shapes as the relative angle between $\mathbf{e}$ and $\boldsymbol{\mu}_{1,2}$ is random. In simulations, one can take into account the orientational average exactly by considering three polarization directions $\mathbf{e}\in\{\mathbf{x},\mathbf{y},\mathbf{z}\}$ for fixed $\boldsymbol{\mu}_{1,2}$ and averaging the corresponding optical coherence dynamics $\mathrm{Tr}\{\mu e^{-iHt}\mu\rho_{th}e^{iHt}\}$ with uniform weighting factors. This can be proved by considering the formal representation of the orientational average of absorption line shapes $\int dR\,\mathrm{Tr}\{(R\mu)e^{-iHt}(R\mu)\rho_{th}e^{iHt}\}$ with $R\mu = \sum_{i=1}^2(\mathbf{e}\cdot R\boldsymbol{\mu}_i)|\varepsilon_i\rangle\langle g| + h.c.$ denoting the transition dipole moment operator where monomer transition dipoles $\boldsymbol{\mu}_i$ are randomly rotated by R in the laboratory frame. By applying the rotation matrix R to $\mathbf{e}$ instead of $\boldsymbol{\mu}_i$, namely $\mathbf{e}\cdot(R\boldsymbol{\mu}_i) = (R^\dagger\mathbf{e})\cdot\boldsymbol{\mu}_i$, one can represent $R^\dagger\mathbf{e} = \cos(\theta)\mathbf{z} + \sin(\theta)\cos(\phi)\mathbf{x} + \sin(\theta)\sin(\phi)\mathbf{y}$ in the spherical coordinate. By substituting $\mathbf{e}$ parameterized by (θ,ϕ) to $\int dR\,\mathrm{Tr}\{(R\mu)e^{-iHt}(R\mu)\rho_{th}e^{iHt}\}$ and then computing the orientational average in the spherical coordinate, one can show that the absorption line shapes of isotropic samples can be computed exactly by considering the three orientations mentioned above[17]. In 2D simulations, discussed in Supplementary Note 8, the orientational average of isotropic samples can be computed exactly by considering a finite number of orientations, similar to absorption simulations[17]. The required orientations can be determined via the concept of spherical t-design which allows the average of any polynomial of the order t over a sphere to be obtained by an average over a discrete set of specific orientations[18,19].

In Supplementary Figure 3a, we consider the single vibrational excitation subspace where all the relative-motion modes are in their vibrational ground states or only one of the 55 modes is singly excited. In simulations, we average the dynamics of optical coherences $\mathrm{Tr}\{\tilde{\mu}e^{-i\tilde{H}t}\tilde{\mu}\rho_{th}e^{i\tilde{H}t}\}$ for 1000 random realizations of site energies, and then multiply $\exp(-\frac{1}{2}G(t))$ induced by center-of-mass modes, as the latter does not depend on static disorder. The eigenvalues and eigenstates of the Hamiltonian $\tilde{H}$ determine, respectively, the transition energies and dipole strengths of vibronic eigenstates. The eigenvalue spectrum depends on the random realizations of site energies, inducing ensemble dephasing of optical coherences. Supplementary Figure 3a shows that the approximate absorption line shape based on the reduced model, shown in red, can quantitatively reproduce the numerically exact absorption line shape of SP, shown in black.

To understand the origin of the three-peak structure in absorption spectrum, we investigate four vibronic eigenstates in the absence of static disorder, including state A appearing at 900 nm, state B at 817 nm, and states C and D at 780 nm, as highlighted in Supplementary Figure 3a. The state A is well described by $|E_-,0\rangle$ where $|E_-\rangle$ is the lower-energy exciton state and $|0\rangle$ denotes the global vibrational ground state. It is notable that the vibronic mixing between $|E_-,0\rangle$ and $|E_+,1_k\rangle$, which can be induced by off-diagonal vibronic couplings, is negligible due to the large energy gap between $|E_-,0\rangle$ and $|E_+,1_k\rangle$, where $|1_k\rangle$ denotes a composite vibrational state with a single vibrational excitation in the k-th mode. For all the eigenstates of $\tilde{H}$, the fidelity with $|E_-,0\rangle$ is shown in green dots, demonstrating that the

mixing with $|E_-,0\rangle$ is negligible for all the eigenstates except for A. The state B is one of the vibronic eigenstates contributing to the vibrational sideband in the 800-850 nm region. The state B is dominated by $|E_-,1_{1008\,\mathrm{cm}^{-1}}\rangle$ with a single vibrational excitation in the $1008\,\mathrm{cm}^{-1}$ mode, which is weakly mixed with quasi-resonant $|E_+,0\rangle$ and multiple $|E_-,1_k\rangle$ states mediated by off-diagonal vibronic couplings. The states C and D, on the other hand, have relatively large fidelity with $|E_+,0\rangle$, as shown in blue dots. Both the states C and D show a strong mixing between $|E_+,0\rangle$ and $|E_+,1_{453\,\mathrm{cm}^{-1}}\rangle$, and relatively weak mixing with multiple $|E_-,1_k\rangle$ states. It is notable that the mixing between $|E_+,0\rangle$ and $|E_+,1_{453\,\mathrm{cm}^{-1}}\rangle$ cannot be directly created by the off-diagonal vibronic couplings proportional to $|E_+\rangle\langle E_-|$ or $|E_-\rangle\langle E_+|$, hinting that it is due to the higher-order interactions mediated by $|E_-,1_k\rangle$ states.

To clarify the origin of the strong mixing between $|E_+,0\rangle$ and $|E_+,1_{453\,\mathrm{cm}^{-1}}\rangle$ in the states C and D, in Supplementary Figure 3b we turn off the higher-order interaction terms in Eq. (39) by neglecting the couplings proportional to $|E_+,1_k\rangle\langle E_-,1_l|$ with $k\neq l$, which induces the transition between exciton states and, at the same time, the exchange of a single vibrational excitation between different modes k and l. Note that the absorption line shapes computed with and without the higher-order interaction terms, shown in Supplementary Figure 3a and b, respectively, are almost identical (see the inset in Supplementary Figure 3b), implying that the higher-order terms are weak couplings. It is also notable that when the higher-order terms are removed from the Hamiltonian $\tilde{H}$, the states A and B are almost unchanged, but the mixing between $|E_+,0\rangle$ and $|E_+,1_{453\,\mathrm{cm}^{-1}}\rangle$ of the states C and D is completely suppressed. These results can be rationalised as follows. In the absence of the higher-order interaction terms, the energy-level of higher-energy exciton state $|E_+,0\rangle$ is increased by $447\,\mathrm{cm}^{-1}$ due to the vibronic mixing with multiple $|E_-,1_k\rangle$ states, leading to the state C in Supplementary Figure 3b, while the vibronic mixing of a vibrationally hot state $|E_+,1_{453\,\mathrm{cm}^{-1}}\rangle$ with $|E_-,0\rangle$ is negligible due to the relatively large energy-gap between them, leading to the state D in Supplementary Figure 3b whose energy-level is almost identical to that of $|E_+,1_{453\,\mathrm{cm}^{-1}}\rangle$. This leads to a small energy-gap $\sim 6\,\mathrm{cm}^{-1}$ between states C and D, which can be strongly mixed even by weak higher-order couplings, as shown in Supplementary Figure 3a. However, the energy-level shifts of the states C and D by the higher-order interaction terms are negligible due to the weak coupling strength, which makes the resulting absorption line shapes insensitive to the presence of the higher-order couplings.

Since the vibronic mixing induced by a weak coupling is likely to be sensitive to energy-level fluctuations induced by environments, such as protein motions, which are not considered in the above reduced model analysis, we performed numerically exact simulations of excitonic coherence dynamics of SP and controlled the vibrational damping rate of the $453\,\mathrm{cm}^{-1}$ modes in order to check if there is a strong vibronic mixing between excitons and $453\,\mathrm{cm}^{-1}$ modes. It is found that the lifetime of the $453\,\mathrm{cm}^{-1}$ modes does not affect excitonic coherence dynamics (not shown here), implying that the vibronic mixing with the $453\,\mathrm{cm}^{-1}$ modes is negligible under actual environments. Therefore we will ignore the higher-order interaction terms, $|E_+,1_k\rangle\langle E_-,1_l|$ with $k\neq l$, in the following approximate simulations of absorption and 2D spectra to avoid weak coupling effects.

Supplementary Figure 3c shows absorption spectra in the absence of off-diagonal vibronic couplings. It is notable that the energy gap between absorption peaks is close to the bare excitonic splitting $\Delta\approx 1300\,\mathrm{cm}^{-1}$ of SP.

Supplementary Figure 3d shows absorption line shapes computed by the reduced vibronic model within double vibrational excitation subspace, which includes doubly excited vibrational states where only the k-th mode is doubly excited, $|2_k\rangle$, or two different modes k and l are singly excited at the same time, $|1_k,1_l\rangle$. We note that the inclusion of doubly excited vibrational states in simulations does not qualitatively change absorption line shapes, but improves the quantitative agreement between approximate and numerically exact absorption line shapes, shown in red and black, respectively.

Supplementary Note 8. Reduced Vibronic Model for Two-Dimensional Electronic Spectra

Here we investigate the nonlinear optical response of SP based on the reduced vibronic model in Supplementary Note 7, which can quantitatively reproduce numerically exact absorption spectra of SP. Contrary to the dynamics of optical coherence of SP, which determines absorption line shape and decays within 200 fs, nonlinear spectroscopic techniques can measure electronic-vibrational dynamics on a picosecond time scale and therefore the noise induced by relative vibrational motions can significantly modify simulated nonlinear spectra. Here we consider the noise originating from the relative motions of low-frequency protein modes and the vibrational damping of 55 intra-pigment modes, which is approximately described by a Lindblad equation. The influence of the center-of-mass motions of the total vibrational environments on 2D electronic spectra (2DES) can be taken into account analytically[4], similar to absorption simulations considered in Supplementary Note 7. We compute rephasing 2D spectra of SP based on the reduced vibronic model in order to demonstrate that oscillatory 2D signals are a mixture of $\Delta'\approx 1800\,\mathrm{cm}^{-1}$ frequency component induced by vibronic coherence $|\psi_+\rangle\langle\psi_-|$ in the electronic excited state manifold, and multiple oscillatory components with frequencies $\omega_k\lesssim 1600\,\mathrm{cm}^{-1}$ originating from vibrational coherences of the 55 intra-pigment modes in the electronic ground state manifold.

As schematically shown in Supplementary Figure 4a, we consider two noise-inducing processes. Firstly, the vibronic coupling between electronic states and low-frequency part $J_l(\omega)$ of the phonon spectral density of SP induces electronic dephasing. In a displaced vibrational basis defined by $U = |g\rangle\langle g| + |E_+\rangle\langle E_+|\prod_{k=1}^{55} D_k\left(\sqrt{\frac{s_k}{2}}A_{++}\right) + |E_-\rangle\langle E_-|\prod_{k=1}^{55} D_k\left(\sqrt{\frac{s_k}{2}}A_{--}\right)$, the electronic dephasing is described by the interaction Hamiltonian in the form

$$\tilde{H}_{ed} = UH_{ed}U^\dagger = U\left(\sum_{i=1}^{2}(-1)^{i-1}|\varepsilon_i\rangle\langle\varepsilon_i|\right)U^\dagger\sum_l f_l(b_l + b_l^\dagger), \tag{41}$$

where b_l represent the relative motions associated with low-frequency phonons including log-normal and marker mode (see Supplementary Note 5). Secondly, the vibrational damping of the relative motions of high-frequency intra-pigment modes is described by the coupling of each mode to an independent secondary bath, modelled by

$$\tilde{H}_{vd} = UH_{vd}U^\dagger = \sum_{k=1}^{55}(b_k^\dagger + b_k)\sum_l g_{k,l}(c_{k,l} + c_{k,l}^\dagger), \tag{42}$$

where $c_{k,l}^\dagger$ denote the bath modes inducing the vibrational damping of the k-th intra-pigment mode b_k. The phonon spectral density $J_{vd,k}(\omega)$ of the k-th secondary bath, characterized by $g_{k,l}$, determines the damping rate of the k-th intra-pigment mode. We consider a Lorentzian spectral density centered at the vibrational frequency ω_k of the intra-pigment mode, so that the coupling strength between intra-pigment mode b_k and environmental modes $c_{k,l}$ is maximized when environmental mode frequencies are resonant with ω_k. The width of the Lorentzian spectral density is taken to be $(50\,\mathrm{fs})^{-1}$, so that the corresponding bath correlation function quickly decays compared to the picosecond timescale of mode damping. It is found that simulated results are not sensitive to the width of the Lorentzian spectral density when it is broader than $(50\,\mathrm{fs})^{-1}$. The amplitude of the Lorentzian spectral density is determined in such a way that the damping rate of the k-th mode is reduced to $(1\,\mathrm{ps})^{-1}$ when the mode is decoupled from electronic states, resulting in a single mode under damping induced by a secondary bath.

To construct a Lindblad equation, the interaction Hamiltonians $\tilde{H}_{ed}$ and $\tilde{H}_{vd}$ are expressed as

$$\tilde{H}_{ed} = A_{ed}\sum_l f_l(b_l^\dagger + b_l), \tag{43}$$

$$\tilde{H}_{vd} = \sum_{k=1}^{55} A_{vd,k}\sum_l g_{k,l}(c_{k,l}^\dagger + c_{k,l}), \tag{44}$$

with $A_{ed} = U\left(\sum_{i=1}^{2}(-1)^{i-1}|\varepsilon_i\rangle\langle\varepsilon_i|\right)U^\dagger$ and $A_{vd,k} = b_k^\dagger + b_k$. We represent the system operators A_{ed} and $A_{vd,k}$ in the eigenbasis of the vibronic system Hamiltonian, $\tilde{H}|\psi_j\rangle = \epsilon_j|\psi_j\rangle$, and define $A_\alpha(\omega) = \sum_{\epsilon'-\epsilon=\omega}\Pi(\epsilon)A_\alpha\Pi(\epsilon')$ for each system operator where $\Pi(\epsilon)$ describes the projection onto the subspace belonging to the eigenvalue ϵ. The noise is described by dissipators in a Lindblad form[20]

$$\frac{d\rho(t)}{dt} = -i[\tilde{H},\rho(t)] + \sum_\alpha\sum_\omega \gamma_\alpha(\omega)[A_\alpha(\omega)\rho(t)A_\alpha^\dagger(\omega) - \frac{1}{2}\{A_\alpha^\dagger(\omega)A_\alpha(\omega),\rho(t)\}], \tag{45}$$

where decoherence rates are determined by $\gamma_\alpha(\omega) = 2\pi J_\alpha(\omega)(n(\omega)+1)$ with $n(\omega) = (\exp(\hbar\omega/k_BT)-1)^{-1}$, which is characterized by the corresponding spectral density, namely $J_\alpha(\omega) = J_{ed}(\omega)$ or $J_\alpha(\omega) = J_{vd,k}(\omega)$ for $k \in \{1,2,3,\cdots,55\}$, and temperature T.

Supplementary Figure 4b displays absorption line shapes of SP computed by HEOM and reduced vibronic models without and with noise, shown in black, red and blue, respectively. Note that the reduced model results with noise are similar to those without noise. This implies that absorption line widths are mainly determined by inhomogeneous broadening and dephasing induced by center-of-mass modes, and the decohering processes described by the Lindblad equation are negligible within the lifetime of optical coherences, which is approximately $\sim 200\,\mathrm{fs}$.

In 2D electronic spectroscopy, a molecular sample is perturbed by three excitation pulses with controlled time delays, which generates third-order optical responses propagating in several phase-matched directions[21,22]. Here we consider rephasing 2D spectra, which can be measured in a particular phase-matched direction[21,22]. The time delay between first and second pulses enables one to resolve excitation wavelengths. The time interval between second and third pulses, called waiting times T, allows for monitoring electronic-vibrational dynamics of molecular systems. In simulations, we consider ground state bleaching (GSB) and stimulated emission (SE) pathways, describing electronic ground and excited state dynamics, respectively[4]. This includes purely vibrational ground state coherences and the excited state coherences between vibronic eigenstates, which lead to oscillatory 2D signals[23–25].

Supplementary Figure 4c shows rephasing 2D spectra at waiting time $T = 0$ in the presence of inhomogeneous broadening. The 2D lineshape, shown as a function of excitation and detection wavelengths, is dominated by a diagonal peak excited and detected at both $\sim 900\,\mathrm{nm}$. The 2D peak location coincides with the transition energy of the main absorption peak A1 marked in Supplementary Figure 4b. To investigate the excited state coherence between vibronic eigenstates $|\psi_-\rangle$ and $|\psi_+\rangle$, inducing absorption peaks A1 and A2, respectively, we consider two cross peaks R12 and R21 highlighted in Supplementary Figure 4c. R12 (R21) is excited at the transition energy of A1 (A2) and then detected at that of A2 (A1).

Supplementary Figure 4d shows the transient of R12 and its Fourier transformation visualizing frequencies present in the transient. It is notable that excited state SE signals, shown in blue, are dominated by oscillations at $\Delta' \approx 1800\,\mathrm{cm}^{-1}$ induced by the vibronic coherence between $|\psi_-\rangle$ and $|\psi_+\rangle$, which are brighter than the other vibronic eigenstates due to excitonic characters. This is contrary to the ground state GSB signals shown in red, which are dominated by low-frequency oscillations at $\sim 125\,\mathrm{cm}^{-1}$, close to the vibrational frequency of the marker mode. This makes the total 2D signals, namely the sum of GSB and SE signals, dominated by the excited state coherence $|\psi_+\rangle\langle\psi_-|$ and the vibrational coherences induced by the marker mode, leading to $\Delta' \approx 1800\,\mathrm{cm}^{-1}$ and $\omega_{\mathrm{sp}} = 125\,\mathrm{cm}^{-1}$ oscillations, respectively.

Supplementary Figure 4e shows the transient of the other cross-peak R21 and its frequency spectrum. Contrary to the case of R12, the oscillation amplitudes of ground state signals, shown in red, are comparable to those of excited state signals at R21. We note that the ground state oscillations consist of multiple components with different frequencies lower than $\sim 1600\,\mathrm{cm}^{-1}$, as the vibrational frequencies ω_k of underdamped intra-pigment modes considered in simulations are lower than $\sim 1600\,\mathrm{cm}^{-1}$ and the Huang-Rhys factors of these modes are of the order of 0.01, which are too small to induce ground state coherences with double vibrational excitation frequencies $\omega_k + \omega_{k'}$. Note that the excited state signals at R21 include $\Delta' \approx 1800\,\mathrm{cm}^{-1}$ oscillations, which are absent in the ground state signals.

Our 2D results demonstrate that long-lived 2D oscillations can be dominated by vibronic coherences even if the frequency of 2D oscillations is not near-resonant with the vibrational frequencies of underdamped modes, namely $\omega_k \lesssim 1600\,\mathrm{cm}^{-1} \ll \Delta' \approx 1800\,\mathrm{cm}^{-1}$. This implies that even if experimentally observed frequency of 2D oscillations is not matched to vibrational frequencies ω_k of intra-pigment modes observed in monomer experiments, there is possibility that the 2D oscillations originate from vibronic coherences induced by multi-mode mixing, rather than from purely electronic coherences[26].

So far we have demonstrated that long-lived oscillations with $\sim 1800\,\mathrm{cm}^{-1}$ frequency appears in 2D spectra when the vibrational damping rates of all the 55 intra-pigment modes are taken to be $\gamma_k = (1\,\mathrm{ps})^{-1}$. In Supplementary Figure 5, we show that the long-lived oscillatory 2D signals originating from a multi-mode vibronic coherence cannot be modelled by coarse-grained spectral densities where only a few intra-pigment modes near-resonant with the excitonic splitting $\Delta \approx 1300\,\mathrm{cm}^{-1}$ of the SP are modelled by narrow Lorentzian spectral densities with a width of $\gamma_k = (1\,\mathrm{ps})^{-1}$, while all the other intra-pigment modes are modelled by broad Lorentzian functions with a width of $\gamma_k = (50\,\mathrm{fs})^{-1}$. In Supplementary Figure 5a, the vibrational damping rates of all the 55 intra-pigment modes are taken to be $\gamma_k = (50\,\mathrm{fs})^{-1}$, leading to a broad spectral density in the high-frequency region, as shown in red. In this case, all the high-frequency 2D oscillations are short-lived, and the $1800\,\mathrm{cm}^{-1}$ component is not visible in the frequency spectrum of 2D signals at the cross-peak R12. In Supplementary Figure 5b, the three intra-pigment modes whose frequencies are close to the excitonic splitting of the SP are modelled by the narrow Lorentzian functions, while all the other intra-pigment modes are approximated by the broad Lorentzian functions, as shown in green. It is notable that the three underdamped vibrational modes lead to long-lived oscillatory 2D signals at their vibrational frequencies $\omega_k \approx \Delta$, but the $1800\,\mathrm{cm}^{-1}$ component is short-lived as the lifetime of a multi-mode vibronic coherence is determined by the overall vibrational damping rate of several tens of the intra-pigment modes involved in a multi-mode vibronic mixing. Even when seven near-resonant intra-pigment modes are modelled by the narrow Lorentzian functions, the long-lived $1800\,\mathrm{cm}^{-1}$ oscillations do not appear in 2D spectra, as shown in blue in Supplementary Figure 5c. These results demonstrate that long-lived multi-mode vibronic coherences cannot be described by conventional vibronic models where only a few near-resonant modes are assumed to be weakly damped, while all the other modes, being relatively off-resonant from the excitonic splitting but participating in the multi-mode vibronic mixing, are severely coarse-grained. We note that the $1800\,\mathrm{cm}^{-1}$ oscillations are not observable in simulations when the off-resonant intra-pigment modes are ignored by taking the corresponding Huang-Rhys factors to be zero, as the multi-mode vibronic energy renormalization, namely $\Delta \approx 1300\,\mathrm{cm}^{-1} \to \Delta' \approx 1800\,\mathrm{cm}^{-1}$ of the SP, cannot be induced by a small number of near-resonant intra-pigment modes, as the Huang-Rhys factors of individual modes are small.

Supplementary Note 9. Population Transfer Dynamics

Here we show that the energy transfer between exciton states can be enhanced by multi-mode vibronic effects. We investigate which intra-pigment modes are excited during the transition from higher to lower energy exciton state in

order to demonstrate that the intra-pigment modes whose vibrational frequencies are near-resonant with a vibronic splitting Δ', instead of a bare excitonic splitting Δ, dominate the excitonic transition. We also compare the exciton transfer dynamics in the presence of highly structured vibrational environments with that of coarse-grained phonon spectral densities where only a few intra-pigment modes whose vibrational frequencies are near-resonant with the excitonic splitting Δ are considered and all the other off-resonant modes are neglected. We show that the transition from higher to lower energy exciton state is more efficient for the multi-mode vibrational environments than for the coarse-grained environments.

In Supplementary Figure 6a and b, we consider a coherent vibronic energy transfer in the SP where exciton states are coupled to 55 intra-pigment vibrational modes. The noise induced by vibrational damping of the intra-pigment modes and vibronic couplings of the excitons to low-frequency phonon environments is ignored, which will be considered later. Starting from an initial state $|E_+, 0\rangle$ with $|E_+\rangle$ denoting a higher-energy exciton state and $|0\rangle$ a global vibrational ground state where all the intra-pigment modes are in their vibrational ground states, we investigate the population dynamics of lower-energy exciton states $|E_-, 0\rangle$, where all the intra-pigment modes are in their vibrational ground states, $|E_-, 1_k\rangle$, where only the k-th intra-pigment mode is singly excited, and $|E_-, 2_k\rangle$ and $|E_-, 1_k, 1_{k'}\rangle$, where only the k-th mode is doubly excited and two different modes k and k' are singly excited at the same time, respectively. In Supplementary Figure 6a, the total population of the lower-energy exciton states is shown in black, which increases rapidly within a sub-100 fs time scale and then fluctuates around 0.75. The total population of $|E_-, 1_k\rangle$, shown in green, is close to the total population of the lower-energy exciton states, shown in black, implying that the single-phonon transitions from $|E_+, 0\rangle$ to $|E_-, 1_k\rangle$ dominate the coherent vibronic energy transfer. The population of $|E_-, 0\rangle$ is close to zero on a picosecond time scale, as shown in blue, but the total population of $|E_-, 2_k\rangle$ and $|E_-, 1_k, 1_{k'}\rangle$ increases slowly, as shown in red, implying that the excitonic transition from higher- to lower-energy exciton state can take place mediated by the creation of two phonons.

To identify which vibrational modes are excited during the excitonic transition, in Supplementary Figure 6b, the populations of the vibrationally cold ($|E_-, 0\rangle$), singly excited ($|E_-, 1_k\rangle$), and doubly excited states ($|E_-, 2_k\rangle$ and $|E_-, 1_k, 1_{k'}\rangle$) at time $t = 1\,\text{ps}$ are shown in blue, green and red dots, respectively, as a function of their detunings from the initial state $|E_+, 0\rangle$, namely $-\Delta$ for $|E_-, 0\rangle$, $\omega_k - \Delta$ for $|E_-, 1_k\rangle$, $\omega_k + \omega_{k'} - \Delta$ for $|E_-, 2_k\rangle$ if $k = k'$ or $|E_-, 1_k, 1_{k'}\rangle$ otherwise. It is notable that the singly excited states $|E_-, 1_k\rangle$ have comparable populations for a wide range of $\omega_k - \Delta$, as shown in green, demonstrating that several tens of the intra-pigment modes can contribute to the excitonic transition. More importantly, the populations of $|E_-, 1_k\rangle$ become higher as the vibrational frequencies ω_k are closer to the vibronic splitting $\Delta' \approx \Delta + 465\,\text{cm}^{-1}$, which is blue-shifted from the bare excitonic splitting Δ due to a multi-mode vibronic mixing. This is in line with the populations of the doubly excited states $|E_-, 2_k\rangle$ and $|E_-, 1_k, 1_{k'}\rangle$, shown in red, which are maximised when the sum of vibrational energy quanta $\omega_k + \omega_{k'}$ is near-resonant with the vibronic splitting Δ'. These results demonstrate that the excitonic transition is governed by the resonance between intra-pigment modes and vibronic splitting Δ', instead of the bare excitonic splitting Δ. These observations can be rationalised as follows. Since the Huang-Rhys factors of individual intra-pigment modes are small, even if a single intra-pigment mode is removed from a full vibronic model, the change in the vibronic splitting Δ' will be small. This implies that the full vibronic model can be decomposed into (i) a multi-mode vibronic model where the excitons are coupled to 54 intra-pigment modes per site and (ii) a perturbation term describing the interaction between excitons and the removed intra-pigment mode with vibrational frequency ω_k, where the contribution of the perturbation term to the 54-mode vibronic model is approximately determined by $\omega_k - \Delta'$.

So far we have considered a purely coherent multi-mode vibronic energy transfer. In the presence of the Lindblad noise induced by vibrational damping of the intra-pigment modes and the vibronic couplings to low-frequency phonon environments at room temperature, the oscillatory features of population dynamics are suppressed, as shown in Supplementary Figure 6c, and the populations of vibronic eigenstates are relaxed to the Boltzmann distribution of a thermal state, as shown in Supplementary Figure 6d.

In Supplementary Figure 7a, the population dynamics of the lower-energy exciton state $|E_-\rangle$ in the absence of the Lindblad noise is shown for several phonon spectral densities, including the full model with 55 undamped intra-pigment modes, shown in black, and coarse-grained models where M intra-pigment modes near-resonant with the excitonic splitting $\Delta \approx 1300\,\text{cm}^{-1}$ of the SP are considered and all the other modes are neglected by taking the corresponding Huang-Rhys factors to be zero: the simulated results for $M = 1, 3, 5$ are shown in blue, green, red, respectively. It is notable that when a single near-resonant mode is considered ($M = 1$), the population of the lower-energy exciton state $|E_-\rangle$ oscillates between 0 and 1, due to a coherent resonant energy transfer between $|E_+, 0_r\rangle$ and $|E_-, 1_r\rangle$ with $|0_r\rangle$ and $|1_r\rangle$ denoting, respectively, the vibrational ground and first excited states of the near-resonant mode. Similarly, when a small number of near-resonant modes are considered ($M = 3, 5$), the population of the lower-energy exciton state $|E_-\rangle$ oscillates with a large amplitude and the population of $|E_-\rangle$ is efficiently transferred back to the higher-energy exciton state $|E_+\rangle$. This is contrary to the multi-mode case, shown in black, where the population of the lower-energy exciton state is increased rapidly on a sub-100 fs time scale and then weakly oscillates around a mean value of ~ 0.8. This implies that multi-mode vibrational environments can induce an ultrafast excitonic transition from $|E_+\rangle$ to $|E_-\rangle$

and then suppresses the back transfer to the higher-energy exciton state $|E_+\rangle$. Even when the Lindblad noise induced by vibrational damping of the intra-pigment modes and low-frequency phonon environments at room temperature is considered in simulations, the energy transfer is more efficient for the multi-mode vibrational environments than for the coarse-grained models. Supplementary Figure 7b shows that the energy transfer rate from $|E_+\rangle$ to $|E_-\rangle$ at early times is enhanced as the number of the intra-pigment modes coupled to the exciton states is increased. In addition, Supplementary Figure 7c shows that the population of the lower-energy exciton state $|E_-\rangle$ is saturated more rapidly for the multi-mode vibrational environments, as the coarse-grained cases show large-amplitude oscillations of the population dynamics, induced by an efficient back transfer to the higher-energy exciton state $|E_+\rangle$. These results demonstrate that the energy transfer dynamics can be enhanced by multi-mode vibronic effects, and we found similar multi-mode vibronic enhancement for various electronic coupling strengths, including $V = 50, 100, 300\,\mathrm{cm}^{-1}$ (not shown here) and $625\,\mathrm{cm}^{-1}$ of the SP considered in Supplementary Figure 7.

Supplementary Note 10. Multi-Mode Vibronic Effects in Site Basis

To explain the influence of multi-mode vibronic effects on absorption spectra, we consider the transition dipole moment operators of PPCs, describing the optical transition between electronic ground and excited states. Within the Franck-Condon approximation, the transition dipole moment operator of a dimeric system is written as

$$\mu = \sum_{i=1}^{2} (\mathbf{e} \cdot \boldsymbol{\mu}_i)(|\varepsilon_i\rangle \langle g| + |g\rangle \langle \varepsilon_i|), \tag{46}$$

where $\mathbf{e}$ is the unit vector in the direction of the electric field inducing absorption, $\boldsymbol{\mu}_i$ the transition dipole moment of site i, and $|g\rangle$ the global electronic ground state. The transition dipole moment operator describes the vertical optical transition from electronic ground to excited states, which launches the vibrational motions of PPCs, inducing 0-0 lines and vibrational sidebands in absorption spectra. The impact of the presence of vibrational modes on the strength of the 0-0 lines can be determined by decomposing the total Hamiltonian H into $H = H_0 + H_I$ where $H_I = V(|\varepsilon_1\rangle \langle \varepsilon_2| + |\varepsilon_2\rangle \langle \varepsilon_1|)$ describes the electronic interaction between monomers, while H_0 determines the energy-level structure of each monomer. The Hamiltonian H_0 is diagonalised by the polaron transformation in site basis, $U = |g\rangle \langle g| + \sum_{i=1}^{2} |\varepsilon_i\rangle \langle \varepsilon_i| D_i$ with unitary displacement operators $D_i = \exp[\sum_{k=1}^{55} \sqrt{s_k}(b_{i,k}^\dagger - b_{i,k})]$, leading to

$$H_0' = \sum_{i=1}^{2} (\varepsilon_i - \lambda_h) |\varepsilon_i\rangle \langle \varepsilon_i| + \sum_{i=1}^{2} \sum_{k=1}^{55} \omega_k b_{i,k}^\dagger b_{i,k}, \tag{47}$$

where $\lambda_h = \sum_{k=1}^{55} \omega_k s_k$ denotes the reorganization energy of the intra-pigment modes. The eigenstates of H_0' include the electronic ground (excited) states $|g, 0\rangle$ ($|\varepsilon_i, 0\rangle$), where all the intra-pigment modes are in their vibrational ground states, and $|g, 1_{i,k}\rangle$ ($|\varepsilon_i, 1_{i,k}\rangle$), where only one mode described by $b_{i,k}$ is singly excited. The transition dipole moment operator in the polaron basis, $\mu' = \sum_{i=1}^{2} (\mathbf{e} \cdot \boldsymbol{\mu}_i)(|\varepsilon_i\rangle \langle g| D_i + h.c.)$, determines the transition dipole strengths between the eigenstates of H_0'. These transition strengths are given by the Franck-Condon factors of uncoupled monomers, such as $\langle g, 0| \mu |\varepsilon_i, 0\rangle = (\mathbf{e} \cdot \boldsymbol{\mu}_i) \exp(-\frac{1}{2} \sum_{l=1}^{55} s_l)$ and $\langle g, 0| \mu |\varepsilon_i, 1_{i,k}\rangle = (\mathbf{e} \cdot \boldsymbol{\mu}_i) \exp(-\frac{1}{2} \sum_{l=1}^{55} s_l)\sqrt{s_k}$, which describe the dipole strengths of the 0-0 transition and the 0-1 transitions (vibrational sideband) in monomer absorption spectrum. This implies that the dipole strength of the 0-0 transition is reduced by the vibronic coupling to the intra-pigment modes by the factor $\exp(-\frac{1}{2} \sum_{l=1}^{55} s_l)$, as the total dipole strength of a monomer is redistributed to 0-n transitions where n vibrational excitations are present amongst the 55 intra-pigment modes. This is in line with the electronic interaction Hamiltonian in the polaron basis, $H_I' = V |\varepsilon_1\rangle \langle \varepsilon_2| \exp[\sum_{k=1}^{55} \sqrt{2 s_k}(b_k^\dagger - b_k)] + h.c.$ with $b_k = (b_{1,k} - b_{2,k})/\sqrt{2}$, that includes the effective coupling between 0-0 transitions, $V_{00} = \langle \varepsilon_1, 0| H_I' |\varepsilon_2, 0\rangle = V \exp(-\sum_{k=1}^{55} s_k) \approx 0.5V$ for WSCP, and the couplings V_{01} between 0-0 and 0-1 transitions, such as $\langle \varepsilon_1, 0| H_I' |\varepsilon_2, 1_{2,k}\rangle$.

To investigate how well the numerically exact absorption spectra of WSCP can be described by the effective coupling V_{00} between 0-0 transitions and how the absorption line shapes are modified by the mixing of 0-0 and 0-1 transitions, we consider a reduced vibronic model where the total Hamiltonian $H' = H_0' + H_I'$ in the polaron basis is numerically diagonalised. We consider a subspace that contains all states involving up to two vibrational excitations of 55 intrapigment modes. This approach enables one to compute the transition energies and dipole strengths of vibronic eigenstates of H' for the cases that the vibronic interaction Hamiltonian H_I' contains only the coupling V_{00} between 0-0 transitions or includes all the vibronic couplings V_{mn} between m-n transitions with $m, n \in \{0, 1, 2\}$ where non-zero values of m describe thermal populations of the intra-pigment modes at finite temperatures. The homogeneous broadening of the vibronic eigenstates is approximately described by a Lindblad equation where the Markovian noise is induced by low-frequency protein modes $J_l^{\mathrm{WSCP}}(\omega)$ and vibrational damping of the intra-pigment

modes. The full reduced model including all the V_{mn} couplings can quantitatively well reproduce the numerically exact absorption line shape of WSCP, as shown in red in Supplementary Figure 8a. Taking into account only V_{00} (dynamic localization) leaves the low-energy part of absorption spectra (above 650 nm) unchanged, as shown in green in Supplementary Figure 8a, but the intensity of the vibrational sideband in a high-energy region is underestimated. This implies that the low-energy part of absorption spectra of WSCP is mainly determined by the effective coupling V_{00} between 0-0 transitions. The deviations in the high-energy part of absorption spectra is due to the mixing of 0-0 and 0-1 transitions, which redistributes transition dipole strengths from 0-0 lines to the vibrational sideband (see Supplementary Note 10 A for details). We note that the reduced model results are scaled by different factors to ensure that the maximum amplitudes of absorption spectra are unity at 656 nm, for a better comparison with numerically exact results. When the full intrapigment spectral density $J(\omega)$, including 55 high-frequency modes, is considered in the conventional line shape theory (see Supplementary Note 10 B for details), the vibronic suppression of the energy-gap between absorption peaks is overestimated by a Lamb shift[20], as shown in blue in Supplementary Figure 8a, and the intensity of the vibrational sideband is significantly underestimated. We note that the Lamb shift originates from a perturbative treatment of vibronic couplings, which is not appropriate to describe the interaction between electronic states and underdamped intrapigment modes where electronic-vibrational correlations are maintained for a long time. These results demonstrate that the multimode vibronic mixing between 0-0 and 0-1 transitions is important for a reliable description of absorption line shapes, especially the intensity of a vibrational sideband, even if the vibronic mixing is not strong enough to modulate the low-energy part of absorption spectra, as shown in red and green in Supplementary Figure 8a. These multimode effects induced by intrapigment modes are robust against variations of the low-frequency vibronic coupling spectrum of protein motions, as shown in Supplementary Figure 8b, where $J_l^{\mathrm{B777}}(\omega)$ is considered instead of $J_l^{\mathrm{WSCP}}(\omega)$.

We note that our numerically exact absorption spectra of WSCP are well matched to the low-energy part of experimental absorption spectra above 640 nm, but the intensity of the computed high-frequency vibrational sideband (with a main peak at 600-610 nm) is stronger than experimental results[1] (not shown here). This implies that the Huang-Rhys factors of the high-frequency intra-pigment modes may be overestimated due to an approximate theory considered in the analysis of experimental FLN data[1], which is in line with the spectral density of WSCP computed by first-principles methods[27]. A more reliable estimation of the Huang-Rhys factors requires numerically exact simulations of the FLN spectra, which is beyond the scope of this work. In addition, the vibrational sideband of Q_y transitions may be vibronically mixed with the zero-phonon lines of Q_x transitions between the electronic ground and second excited states of the pigments[28], which is not considered in this work and deserves separate investigation.

A. Multi-Mode Vibronic Mixing of 0-0 and 0-1 Transitions and Lamb Shift

Here we show the influence of multi-mode vibronic mixing of 0-0 and 0-1 transitions of WSCP and Lamb shift on absorption line shapes of WSCP in more detail.

In reduced model simulations of absorption, we numerically diagonalize the total Hamiltonian $H' = H_0' + H_I'$ in the polaron basis within double vibrational excitation subspace and compute the energy-levels and transition dipole strengths of vibronic eigenstates. The broadening of absorption peaks is modelled by the static disorder in site energies and homogeneous broadening induced by the coupling to low-frequency protein motions and the vibrational damping of intra-pigment modes. The center-of-mass motions of the protein modes are treated non-perturbatively, while the relative motions of the protein modes are considered approximately by using a Lindblad equation (see Supplementary Note 8). In addition, the vibrational damping of local intra-pigment modes is considered within the Lindblad formalism where each intra-pigment mode is coupled to an independent secondary bath in a thermal state, similar to the reduced model for SP (see Supplementary Note 8). We assume that the intra-pigment modes are initially in a thermal state at temperature $T = 77\,\mathrm{K}$.

In Supplementary Figure 9a, we consider only the V_{00} coupling between 0-0 transitions and neglect all the other couplings in H_I' (dynamic localization). Numerically exact absorption spectrum of WSCP is shown in a black dashed line, while approximate absorption line shape is shown in a red solid line. The contributions of 0-n, 1-n and 2-n transitions to the total approximate absorption spectra are shown in blue solid, blue dashed and blue dotted lines, respectively. The 1-1 transitions lead to a single peak at 659 nm due to the absence of the interaction between $|\varepsilon_1, 1_{j,k}\rangle$ and $|\varepsilon_2, 1_{j',k'}\rangle$. Note that the total approximate absorption line shape shown in red is not well matched to numerically exact results shown in black.

In Supplementary Figure 9b, we consider the interaction between local electronic excitations when associated vibrational states are identical, such as the coupling between $|\varepsilon_1, n_{j,k}\rangle$ and $|\varepsilon_2, n_{j,k}\rangle$ with $n_{j,k} \in \{0, 1, 2\}$, and that between $|\varepsilon_1, 1_{j,k}, 1_{j',k'}\rangle$ and $|\varepsilon_2, 1_{j,k}, 1_{j',k'}\rangle$ where different modes $b_{j,k}$ and $b_{j',k'}$ are singly excited at the same time. This modifies the absorption line shape of 1-n transitions, leading to two absorption peaks in zero-phonon line region above 650 nm, similar to 0-n transitions. To clarify the energy-gap between 0-0 transitions, the transition dipole

strengths of all 0-n transitions are shown in yellow triangles as a function of transition energies, where the splitting between 0-0 transitions is $\Delta' = 2V_{00} \approx 142\,\mathrm{cm}^{-1}$. Note that approximate absorption spectra are still not well matched to numerically exact results.

In Supplementary Figure 9c, we now consider the full H'_I, containing the couplings between local electronic excitations with different vibrational states. This leads to a multi-mode vibronic mixing between 0-0 and 0-1 transitions, which redistributes the transition dipole strengths of the bright 0-0 transition at 655.5 nm and relatively dark 0-1 transitions around 650 nm (compare yellow triangles in Supplementary Figure 9b and c). This makes the main absorption peak at 655.5 nm darker, and the vibrational sideband around 650 nm brighter. As a result, approximate absorption line shape shown in red is quantitatively well matched to numerically exact absorption spectrum shown in black, although the approximate results underestimate absorption in the low-energy region above 662 nm. This implies that the energy-gap $\Delta' \approx 134\,\mathrm{cm}^{-1}$ between absorption peaks in zero-phonon line region is underestimated when compared to numerically exact results.

So far we have not included the Lamb shift in reduced model simulations. Within the Lindblad formalism[20], the energy-level shift induced by the interaction with environmental degrees of freedom is described by

$$H_{LS} = \sum_{\omega}\sum_{\alpha} S_\alpha(\omega) A_\alpha^\dagger(\omega) A_\alpha(\omega) \tag{48}$$

$$= -\sum_j |\psi_j\rangle\langle\psi_j| \sum_\alpha \lambda_\alpha |\langle\psi_j|A_\alpha|\psi_j\rangle|^2 + \sum_{\epsilon_k\neq\epsilon_j} |\psi_k\rangle\langle\psi_k| \sum_\alpha S_\alpha(\epsilon_k-\epsilon_j)|\langle\psi_j|A_\alpha|\psi_k\rangle|^2, \tag{49}$$

where $|\psi_j\rangle$ represent the vibronic eigenstates of the total Hamiltonian $UHU^\dagger = H_0 + H_I$, satisfying $UHU^\dagger|\psi_j\rangle = \epsilon_j|\psi_j\rangle$, A_α noise operators describing the coupling to low-frequency protein motions or vibrational damping of intra-pigment modes, $S_\alpha(\Delta') = \mathrm{P}\int_{-\infty}^{\infty} d\omega (J_\alpha(\omega)(n(\omega)+1) + J_\alpha(-\omega)n(-\omega))/(\Delta'-\omega)$ and $\lambda_\alpha = \int_0^\infty d\omega J_\alpha(\omega)/\omega$ with $J_\alpha(\omega)$ denoting the environmental spectral density describing the noise process associated with A_α (see Supplementary Note 8). Supplementary Figure 9d shows that when the Lamb shift is included in simulations, the energy-gap $\Delta' \approx 134\,\mathrm{cm}^{-1}$ renormalised by the intra-pigment modes is increased to $\Delta'' \approx 188\,\mathrm{cm}^{-1}$. The difference between approximate and numerically exact absorption line shapes can be further reduced by introducing a small empirical pure dephasing rate $\gamma_{pd} = (2\,\mathrm{ps})^{-1}$, as shown in a green solid line. Within the Lindblad formalism, the phonon spectral density of protein motions of WSCP does not induce a pure dephasing process, as log-normal distribution functions are super-Ohmic, which may underestimate the pure dephasing effect in numerically exact simulations.

These results demonstrate that the interaction between 0-0 transitions is significantly suppressed by a local vibronic coupling to high-frequency intra-pigment modes, and a multi-mode vibronic mixing of 0-0 and 0-1 transitions can modify absorption line shapes even if the mixing is not strong enough to change the energy-gap between absorption peaks originating from 0-0 transitions. This is similar to the properties of bare exciton states where the energy-gap between excitons is close to the difference in site energies, $|\varepsilon_2 - \varepsilon_1|$, when the electronic coupling V between monomers is sufficiently weak, but such a weak coupling can induce notable redistribution of oscillator strengths, leading to large difference in transition dipole strengths of exciton states[29]. The Lamb shift induced by low-frequency protein motions can increase or decrease the energy-gap between absorption peaks, similar to the multi-mode vibronic mixing induced by the intra-pigment modes, hinting that characterisation of the environmental structures of photosynthetic pigment-protein complexes is essential to understand the optical responses of these systems. We note that in photosynthetic systems, pigments may be coupled to non-identical vibrational environments, characterised by different spectral densities. Even in this case, one can generalise our approach and show that the effective coupling between 0-0 transitions of sites i and j is given by $V_{ij}\exp(-\frac{1}{2}\sum_k(s_{i,k}+s_{j,k}))$ where V_{ij} denotes the bare electronic coupling between sites i and j of a multi-chromophoric system, and $s_{i,k}$ the Huang-Rhys factors of the intra-pigment modes locally coupled to site i.

B. Line Shape Theory

Here we consider conventional line shape theory based on second order cumulant expansion where absorption line shape of a dimer is approximately described by

$$\sum_{j=\pm} |\boldsymbol{\mu}_{E_j}|^2 \int_{-\infty}^{\infty} dt \exp[i(\omega - E_j - E_{LS,j})t + G_j(t) - G_j(0) - (\gamma_{j\to k}/2 + \gamma_{pd})|t|],$$

where $\boldsymbol{\mu}_{E_j}$ and E_j denote the transition dipole moment vector and energy-level, respectively, of an exciton state $|E_j\rangle$, defined by $H_e|E_j\rangle = E_j|E_j\rangle$, and $G_j(t) = \sum_{i=1}^{2}|\langle\varepsilon_i|E_j\rangle|^4 G(t)$ with $G(t)$ characterised by the full spectral density

$J(\omega)$ of WSCP, including both low-frequency protein and high-frequency intra-pigment modes

$$G(t) = \int_0^\infty d\omega\, \omega^{-2}[J(\omega)(n(\omega)+1)e^{-i\omega t} + J(\omega)n(\omega)e^{i\omega t}].$$

Here γ_{pd} is an empirical pure dephasing rate, while $\gamma_{j\to k}$ represents an incoherent exciton population transfer rate from $|E_j\rangle$ to $|E_k\rangle$

$$\gamma_{\pm\to\mp} = 2\pi \sum_{i=1}^{2} |\langle E_+|\varepsilon_i\rangle\langle\varepsilon_i|E_-\rangle|^2 (J(\pm\Delta)(n(\pm\Delta)+1) + J(\mp\Delta)n(\mp\Delta)),$$

with $J(\omega)=0$ for $\omega<0$, $\Delta = E_+ - E_- > 0$ and $n(\omega) = (\exp(\omega/k_B T)-1)^{-1}$. The Lamb shift $E_{LS,j}$ describes the energy-level shift of an exciton state $|E_j\rangle$ due to the interaction with the full vibrational environments

$$E_{LS,\pm} = -\sum_{i=1}^{2} |\langle\varepsilon_i|E_\pm\rangle|^4\lambda + \sum_{i=1}^{2} |\langle E_+|\varepsilon_i\rangle\langle\varepsilon_i|E_-\rangle|^2\, \mathrm{P}\int_{-\infty}^{\infty} d\omega\, \frac{J(\omega)(n(\omega)+1) + J(-\omega)n(-\omega)}{\pm\Delta - \omega}, \tag{50}$$

with P denoting the Cauchy principal value and $\lambda = \int_0^\infty d\omega J(\omega)/\omega$, which is a special case of Eq. (49). When the full phonon spectral density of WSCP is considered, the energy-gap between excitons is reduced from a bare excitonic splitting $\Delta \approx 2V = 280\,\mathrm{cm}^{-1}$ to $\Delta' \approx 102\,\mathrm{cm}^{-1}$ by the Lamb shift. This implies that the Lamb shift dominated by high-frequency intra-pigment modes suppresses the energy-gap between exciton states, although the Markov approximation considered in the derivation of the Lamb shift is not appropriate to describe the long-lived correlations between excitons and underdamped intra-pigment modes. It is notable that the suppression of the excitonic splitting is over-estimated by the conventional line shape theory, which cannot reproduce numerically exact absorption spectra of WSCP (see Supplementary Figure 8).

Supplementary References:

[1]J. Pieper, M. Rätsep, I. Trostmann, H. Paulsen, G. Renger, and A. Freiberg, J. Phys. Chem. B **115**, 4042 (2011).
[2]V. Zazubovich, I. Tibe, and G. J. Small, J. Phys. Chem. B **105**, 12410 (2001).
[3]J. Lim, M. Tame, K. H. Yee, J.-S. Lee, and J. Lee, New J. Phys. **16**, 018001 (2014).
[4]S. Mukamel, *Principles of nonlinear optical spectroscopy* (Oxford University Press, 1995).
[5]J. Prior, A. W. Chin, S. F. Huelga, and M. B. Plenio, Phys. Rev. Lett. **105**, 050404 (2010).
[6]A. W. Chin, Á. Rivas, S. F. Huelga, and M. B. Plenio, J. Math. Phys. **51**, 092109 (2010).
[7]D. Tamascelli, A. Smirne, J. Lim, S. F. Huelga, and M. B. Plenio, Phys. Rev. Lett. **123**, 090402 (2019).
[8]A. W. Chin, J. Prior, R. Rosenbach, F. Caycedo-Soler, S. F. Huelga, and M. B. Plenio, Nat. Phys. **9**, 113 (2013).
[9]D. Tamascelli, A. Smirne, S. F. Huelga, and M. B. Plenio, Phys. Rev. Lett. **120**, 030402 (2018).
[10]T. Renger and R. A. Marcus, J. Chem. Phys. **116**, 9997 (2002).
[11]D. Tamascelli, R. Rosenbach, and M. B. Plenio, Phys. Rev. E **91**, 063306 (2015).
[12]L. Kohn, F. Tschirsich, M. Keck, M. B. Plenio, D. Tamascelli, and S. Montangero, Phys. Rev. E **97**, 013301 (2018).
[13]T.-C. Dinh and T. Renger, J. Chem. Phys. **142**, 034104 (2015).
[14]A. Kell, X. Feng, M. Reppert, and R. Jankowiak, J. Phys. Chem. B **117**, 7317 (2013).
[15]A. Khmelnitskiy, T. Reinot, and R. Jankowiak, J. Phys. Chem. B **123**, 852 (2019).
[16]M. Reppert, A. Kell, T. Pruitt, and R. Jankowiak, J. Chem. Phys. **142**, 094111 (2015).
[17]J. Lim, C. M. Bösen, A. D. Somoza, C. P. Koch, M. B. Plenio, and S. F. Huelga, Phys. Rev. Lett. **123**, 233201 (2019).
[18]B. Bajnok, European J. Combin. **12**, 377 (1991).
[19]J. Makino, J. Comput. Phys. **151**, 910 (1999).
[20]H. P. Breuer and F. Petruccione, *The theory of open quantum systems* (Oxford University Press, 2000).
[21]D. M. Jonas, Annu. Rev. Phys. Chem. **54**, 425 (2003).
[22]T. Brixner, T. Manal, I. V. Stiopkin, and G. R. Fleming, J. Chem. Phys. **121**, 4221 (2004).
[23]M. B. Plenio, J. Almeida, and S. F. Huelga, J. Chem. Phys. **139**, 235102 (2013).
[24]V. Tiwari, W. K. Peters, and D. M. Jonas, Nat. Chem. **6**, 173 (2014).
[25]J. Lim, D. Paleček, F. Caycedo-Soler, C. N. Lincoln, J. Prior, H. von Berlepsch, S. F. Huelga, M. B. Plenio, D. Zigmantas, and J. Hauer, Nat. Comm. **8**, 7755 (2015).
[26]D. Hayes, G. B. Griffin, and G. S. Engel, Science **340**, 1431 (2013).
[27]A. M. Rosnik and C. Curutchet, J. Chem. Theory Comput. **11**, 5826 (2015).
[28]J. R. Reimers, Z.-L. Cai, R. Kobayashi, M. Rätsep, A. Freiberg, and E. Krausz, Sci. Rep. **3**, 2761 (2013).
[29]T. Renger, J. Voigt, and V. May, J. Phys. Chem. **100**, 15654 (1996).
